# Supplementary material for: Biophysical modeling reveals the transcriptional regulatory mechanism of Spo0A, the master regulator in starving Bacillus subtilis
Source: mSystems. 2025 Apr 29;10(5):e00072-25. doi: 10.1128/msystems.00072-25 (PMC12090719; doi:10.1128/msystems.00072-25)
Supplement: Supplemental Material — Theoretical methods and supplemental figures and tables. [file msystems.00072-25-s0001.pdf]

SUPPLEMENTAL INFORMATION FOR

Biophysical modeling reveals the transcriptional regulatory mechanism of Spo0A, the master regulator in starving *Bacillus subtilis*

Yujia Zhang[1], Cristina S.D. Palma[1], Zhuo Chen[1], Brenda Zarazúa-Osorio[2 3], Masaya Fujita[2] and Oleg Igoshin[1,4]*

[1]Department of Bioengineering, Rice University, Houston, Texas, USA

[2]Department of Biology and Biochemistry, University of Houston, Houston, Texas, USA

[3]Present address: Department of Psychological Sciences, Rice University, Houston, Texas, USA

[4]Departments of Chemistry and of Biosciences, Center for Theoretical Biological Physics, and Rice Synthetic Biology Institute, Rice University, Houston, Texas, USA

*Corresponding author: Oleg Igoshin e-mail: igoshin@rice.edu

This PDF includes:

Theoretical Methods.. S2

Supplementary Tables.. S31

Supplementary Figures.. S42

References.. S53

Theoretical Methods

Contents

(I) Model the fraction of DNA bound by 0A~P S3

(i.1) Computing the fraction of bound DNA using the law of mass action under equilibrium.. S3

(i.2) Augmenting EMSA data S3

(i.3) Fitting the mathematical expression to EMSA data S4

(i.4) Developing a general statistical thermodynamic model and predicting the fraction of bound DNA for strains with multiple binding sites S5

(II) Biophysically model promoter activity for DNA with a single promoter S7

(ii.1) Binding probability, $P\alpha$ S8

(ii.2) Maximum initiation rate, $vmax$ S10

(III) Biophysically model promoter activity for DNA with two promoters S11

(IV) Biophysical models with different types of transcription control S14

(iv.1) Biophysical model with purely thermodynamic control S14

(iv.1.a) For a single-promoter mutant strain S14

(iv.1.b) For a double-promoter mutant strain S15

(iv.2) Biophysical model with purely kinetic control S18

(iv.2.a) For a single-promoter mutant strain S18

(iv.2.b) For a double-promoter mutant strain S20

(iv.3) Model with mixed control S22

(V) Constrain the biophysical gene regulatory model with EMSA predicted parametersS23

(VI) Compute 0A box occupancy probability using best-fit kinetic model parameters... S24

(VII) Bayesian Information Criterion for differentially constrained models S25

(VIII) Model-predicted regulatory role of 0A boxes determined from $vmax$ and $veffective$ S25

(viii.1) for single-promoter strains S26

(viii.2) for double-promoter strains S27

(IX) Find RNAP holoenzyme dynamics common to *spo0A* and non-*spo0A* promoters.. S28

(I) Model the fraction of DNA bound by 0A~P

In this section, we describe the data processing of the electrophoresis mobility shift assay (EMSA) used to examine the properties of 0A boxes and constrain the biophysical model.

(i.1) Computing the fraction of bound DNA using the law of mass action under equilibrium

The process of a particular 0A box being bound by 0A~P can be described according to the reaction scheme *Eqn. I. 1,* with the forward reaction rate k_1 and backward reaction k_2:

$$0A_{box}^{free} + N \cdot 0A{\sim}P \rightleftarrows 0A_{box}^{bound} \qquad \textit{Eqn. I. 1}$$

Here, as in the main manuscript, $0A_{box}^{free/bound}$ stands for free/bound binding site conformation, respectively, and *N* is the number of free 0A~P molecules that bind to the free site. In accordance with the mass-action equilibrium law, one can write the dissociation constant K_d as:

$$K_d = \frac{k_2}{k_1} = \frac{\left[0A_{box}^{free}\right] \times [0A{\sim}P]^N}{\left[0A_{box}^{bound}\right]} \qquad \textit{Eqn. I. 2}$$

Assuming the total concentration of the binding site to be 1, the sum of bound and unbound form can be written as:

$$1 = \left[0A_{box}^{free}\right] + [0A_{box}^{bound}] \qquad \textit{Eqn. I. 3}$$

Then, by combining *Eqn. I. 2* and *Eqn. I. 3,* the concentration of bound binding sites can be written as:

$$[0A_{box}^{bound}] = \frac{[0A{\sim}P]^N/K_d}{1 + [0A{\sim}P]^N/K_d} = \frac{[0A{\sim}P]^N}{K_d + [0A{\sim}P]^N} = \frac{[0A{\sim}P]^N}{\left(K_{half}\right)^N + [0A{\sim}P]^N} \qquad \textit{Eqn. I. 4}$$

We chose to report binding affinity in the same unit of the 0A~P concentration. Therefore, we let $K_d = \left(K_{half}\right)^N$, where K_{half} is the 0A~P concentration resulting in a half-maximal level (i.e., the probability that a 0A box is bound is 50%).

(i.2) Augmenting EMSA data

To quantify the fraction of DNA bound to 0A~P, we performed gel electrophoresis mobility shift assay (EMSA) for DNA fragments with mutagenesis combinations of the 0A boxes (1). These

fragments include those with single boxes (0A1, 0A2, and 0A3) and those with multiple boxes (0A12, 0A13, 0A23, 0A123). For each DNA fragment, the experiment was conducted over 11 concentrations of 0A~P (0 to 2 *μM*, at an interval of 0.2) with *M* biological replicates, with $M \in \{2,3,4\}$.

To model the fraction of bound DNA ($0A_{box}^{bound}$) for strains with only one 0A box ("1", "2", "3", denoting the presence of 0A boxes), we performed the augmentation protocol for these three DNA fragments. The protocol is as follows:

1. From the biological replicate data, we compute the mean and standard deviation of the fraction of bound DNA, at each 0A~P concentration. We then randomly sampled from a Gaussian distribution with the same mean and standard deviation to obtain the augmented data point at each corresponding 0A~P concentration.

2. Given that, for each DNA fragment, there are a total of $11 \times M$ experimental data points before augmentation. Using the random sampling method above, we also sampled $11 \times M$ data points, for each DNA fragment.

3. Combining augmented data points for all three types of DNA fragment, this leads to $\sum_{s=1}^{s=3} 11 \times M_s$ newly generated data points, where M_s is the number of replicate experiments for each type of DNA fragment "s". We consider these many data points as one augmented dataset.

4. We repeated the protocol 1000 times, leading to 1000 augmented EMSA datasets in total.

(i.3) Fitting the mathematical expression to EMSA data

Given known concentrations of 0A~P, we want to solve for the half-saturation constant (K_{half}) and the number of 0A~P molecules (N). To this end, we fit *Eqn. I. 4* to a single augmented dataset of the single-0A box measurements. The optimization error between model prediction and augmented empirical EMSA data (E_{EMSA}) is defined as:

$$E_{EMSA} = \frac{1}{\sum_{s=1}^{s=3} 11 \times M_s} \sum_{s=1}^{s=3} \sum_{r=1}^{r=M_s} \sum_{t=1}^{t=11} \left(P_{bound(EMSA)_{s,r,t}} - P_{bound(Model)_{s,r,t}}\right)^2 \qquad \textit{Eqn. I. 5}$$

Where, s is the type of DNA fragment ("1", "2", "3"), r is the particular replicate out of M_s total replicates for each DNA fragment ($M_s \in \{2,3,4\}$ depending on s), and t is the concentration of 0A~P unique to each measurement. $P_{bound(EMSA)_{s,r,t}}$ and $P_{bound(Model)_{s,r,t}}$ is the experimental data and model-generated data, at a specific s, r, t, respectively.

A total of 1000 sets of K_d parameters, including K_{half} for each 0A box ($K_{half_1}, K_{half_2}, K_{half_3}$) are obtained by fitting *Eqn. I. 4* to all 1000 augmented datasets, one at a time. The model fitted to each augmented dataset is optimized at least 20 times using the MATLAB Particle Swarm Optimization algorithm (2). The mean with a 95% confidence interval in each optimized K_{half} parameter across 1000 augmented sets is shown in Figure 4B. We also examined the model fits by examining different possible values of N, and only $N = 4$ fits the best (Table S2).

(i.4) Developing a general statistical thermodynamic model and predicting the fraction of bound DNA for strains with multiple binding sites

In the previous section i.1, we showed that the concentration of bound DNA can be computed using the law of mass action under equilibrium, assuming the total concentration of DNA is 1. This approach is equivalent to computing the probability of DNA being bound (P_{bound}) using equilibrium statistical thermodynamics.

Specifically, to compute P_{bound}, we divide the statistical weight representing 0A~P bound by the sum of the statistical weights representing all possible system states (3, 4). For a DNA fragment with only one 0A box present, only two possible system states are possible: 0A~P bound to the 0A box, or 0A~P free. As a result, the probability of a DNA fragment with one 0A box being bound by 0A~P *($P_{bound,\ one\ box}$)* is given by *Eqn. I. 6*, where the statistical weight of each state depends on the binding energy at a particular 0A box (G_i) and the cooperativity (N) (5, 6). The unbound state is assumed to have zero binding energy.

$$\mathrm{P_{bound,one\ box}} = \frac{[\mathrm{0A{\sim}P}]^{\mathrm{N}} \exp(-\mathrm{G_i})}{\underbrace{1}_{\substack{\text{Statistical weight}\\ \text{of unbound state}}} + \underbrace{[\mathrm{0A{\sim}P}]^{\mathrm{N}} \exp(-\mathrm{G_i})}_{\substack{\text{Statistical weight}\\ \text{of bound state}}}} \qquad \textit{Eqn. I. 6}$$

Noteworthy, comparing *Eqn. I. 4* and *Eqn. I. 6*, we see that, for a 0A box *'i':*

$$K_{d_i} = \exp\,(G_i) \text{ and } K_{half_i} = \exp\left(\frac{G_i}{N}\right) \qquad \textit{Eqn. I. 7}$$

Where $i \in [1,2,3]$

To understand why this is true intuitively, consider the case where, in *Eqn. I. 6,* a more negative binding energy increases the statistical weight of the bound state to increase the probability of DNA being bound. When the binding energy is more negative, K_d become smaller, suggesting that the forward reaction (DNA is bound) is favored and that the binding affinity is higher.

Next, we derive statistical thermodynamic expressions to compute the probability of DNA being bound by 0A~P for DNA fragments with multiple 0A boxes. For DNA fragments with two 0A boxes, box *i* and *j*, there are four possible states the DNA is bound by 0A~P: 1) neither *i* nor *j* bound; 2) *i* bound; 3) *j* bound; 3) *i* and *j* bound. As such, the cumulative probability where at least one 0A box is bound ($P_{bound,two\ boxes}$) can be written as:

$$P_{bound,two\ boxes} = 1 - \frac{1}{\underbrace{1}_{Neither\ Bound} + \underbrace{[0A{\sim}P]^N exp(-G_i)}_{i\ Bound} + \underbrace{[0A{\sim}P]^N exp(-G_j)}_{j\ Bound} + \underbrace{[0A{\sim}P]^{2N} exp(-G_i - G_j - G_{ij})}_{Both\ Bound}} \qquad \textit{Eqn. I. 8}$$

Where, G_i and G_j are the binding energies of each 0A box, and G_{ij} is the interaction energy between two bound transcriptional factors (also known as "secondary interaction energy"). The variable *N* represents the cooperativity level between 0A~P molecules. The underlying brackets indicate the type of configuration represented by the Boltzmann weight.

For DNA fragments with three 0A boxes, box *i*, *j*, and *k*, there are 2^3 possible configurations that the DNA fragment is bound by 0A~P. Similarly, one can calculate the cumulative probability where at least one 0A box is bound ($P_{bound,three\ boxes}$) as:

$$P_{bound,three\ boxes} = 1 - \frac{1}{Z} \qquad \textit{Eqn. I. 9}$$

Where, $Z = Z_1 + Z_2 + Z_3 + Z_4$. The decomposition of Z_1, Z_2, Z_3 and Z_4 is shown below:

$$Z_1 = 1 + [0A{\sim}P]^{N_{0A}} exp(-G_i) + [0A{\sim}P]^{N_{0A}} exp(-G_j) + [0A{\sim}P]^{N_{0A}} exp(-G_k) \quad \textit{Eqn. I. 10}$$

$$Z_2 = [0A{\sim}P]^{2N_{0A}} exp(-G_i - G_j - G_{ij}) + [0A{\sim}P]^{2N_{0A}} exp(-G_i - G_k - G_{ik}) + [0A{\sim}P]^{2N_{0A}} exp(-G_j - G_k - G_{jk}) \quad \textit{Eqn. I. 11}$$

$$Z_3 = [0A{\sim}P]^{3N_{0A}} exp(-G_i - G_j - G_k - G_{ij} - G_{ik} - G_{jk} - G_{ijk}) \quad \textit{Eqn. I. 12}$$

Noteworthy, in *Eqn. I. 9,* in addition to secondary energy between every pair of 0A boxes (G_{ij}), the tertiary interaction energy (G_{ijk}) when all three 0A boxes are bound is also considered. Particularly, Z_1 is the sum of statistical weight for configurations with only one 0A box occupied; Z_2 represents all statistical weights for configurations with two 0A boxes occupied. Z_3 is the statistical weight of the configuration with all three boxes being occupied.

We then use each set of model-optimized K_d parameters from section i.3 to compute the probability of DNA being bound, for strains with two or more 0A boxes ("12", "13", "23", and "123"). To start with, we convert K_d parameters to the binding energy G based on *Eqn. I. 7*. Then, assuming no secondary or tertiary energy, i.e., $G_{ij} = 0$ and $G_{ijk} = 0$, we compute the probability of DNA bound using *Eqn. I. 8* and *Eqn. I. 9*. The predicted results are shown in Supplementary Figure S2.

(II) Biophysically model promoter activity for DNA with a single promoter

The single-promoter model framework is applied to strains with only P*v* present ("P*v* strains") and only P*s* present ("P*s* strains").

For a DNA fragment with a single promoter and with $n-1$ ligand sites, binding of transcription factors (TFs) and transcriptional machinery results in 2^n possible unique ways in which these *n* sites can be occupied. Each possible way is defined as a "configuration" (α), which is represented using binary variables (σ):

$$\alpha = [\sigma_1, \sigma_2, \sigma_3, \sigma_{pro}] \quad \textit{Eqn. II. 1}$$

Where $\sigma_i = 1$ denotes the site being occupied, and 0 otherwise. In this specific model, the variables $\sigma_1, \sigma_2, \sigma_3$ represents bound/unbound 0A1-3 box, respectively. The variable σ_{pro} represents bound/unbound promoter by RNAP.

Next, for the configurations that can transcribe, i.e., those with σ_{pro} = 1, the effective transcription rate ($v^{effective}$) is modeled as the product of RNAP binding probability (P) and the maximum transcription initiation rate (v_{max}) (6–9). The effective transcription rate of each mutant strain (*MS*) considers the contributions from all promoter-bound configurations, and is therefore calculated as follows:

$$v_{MS_{one\,promoter}}^{effective} = \sum_{\alpha \in \alpha_T} v_{max_\alpha} P_\alpha \qquad \textit{Eqn. II. 2}$$

Where, $v_{MS_{one\,promoter}}^{effective}$, is the effective transcription rate of a mutant strain with a single promoter (mutant strain, or 'MS', can be '1', '2', '3', …, '123', and 'none', depending on the type of strain used in the experiment). Variable, α_T, is the number of possible configurations with promoter bound, i.e., α_T = $[\sigma_1, \sigma_2, \sigma_3, \sigma_{pro} = 1]$.

Assuming both reactions happen at thermodynamic equilibrium, in the below sections, we expand P_α and $vmax_\alpha$ using dimensionless energy landscape parameters (Figure S11) (5, 6).

(ii.1) Binding probability, P_α

The binding probably of RNAP to the promoter can be written as the ratio between the Boltzmann weight (BW) of a particular configuration (α_T) and the sum of Boltzmann weights over all configurations (also known as the partition function) (5–7, 10), as shown in *Eqn.II.3*:

$$P_{\alpha \in \alpha_T} = \frac{BW_{\alpha \in \alpha_T}}{\sum BW_\alpha} = \frac{BW_{\alpha \in \alpha_T}}{\sum BW_{\alpha \in \alpha_T} + \sum BW_{\alpha \in \alpha_{TF}}} \qquad \textit{Eqn. II. 3}$$

Here, α_{TF} corresponds to the configurations where RNAP is not bound to the promoter (i.e., $\alpha_{TF} = [\sigma_1, \sigma_2, \sigma_3, \sigma_{pro} = 0]$). The Boltzmann weight of each configuration (BW_α) depends on the free energy of the configuration, the concentration of the bound ligands, and the cooperativity of the ligands. As such, one can further expand *Eqn. II.3* as:

$$P_{\alpha \in \alpha_T} = \frac{exp\left(-G^\circ_{\alpha \in \alpha_T}\right) \times [0A{\sim}P]^{N_{0A}} \times [R]^{N_{pol}}}{\sum_\alpha exp(-G^\circ_\alpha) \times [0A{\sim}P]^{N_{0A}} \times [R]} \qquad \textit{Eqn. II. 4}$$

Where, $[R]$ and $[0A{\sim}P]$ are the concentration of RNAP and Spo0A~P, respectively. Variable $G^{\circ}{}_{\alpha}$ is the free energy of configuration α. The variables N_{0A} and N_{pol} are the number of bound Spo0A~P molecules and the number of bound RNAP, respectively. We assume $N_{pol} = 1$, given that not more than one RNAP can bind simultaneously to a single promoter region. N_{0A} is assumed to be multiples of 4 based on the estimation of EMSA data (Result Section 2.2; Supplementary Section I).

Further, the denominator of *Eqn. II.4*, can be decomposed into configurations with promoter bound (α_T) and promoter unbound by RNAP (α_{TF}):

$$P_{\alpha\in\alpha_T} = \frac{exp\left(-G^{\circ}{}_{\alpha\in\alpha_T}\right) \times [0A{\sim}P]^{N_{0A}} \times [R]^{N_{pol}}}{\sum_{\alpha\in\alpha_T} exp\left(-G^{\circ}{}_{\alpha\in\alpha_T}\right) \times [0A{\sim}P]^{N_{0A}} \times [R] + \sum_{\alpha\in\alpha_{TF}} exp\left(-G^{\circ}{}_{\alpha\in\alpha_{TF}}\right) \times [0A{\sim}P]^{N_{0A}}} \qquad \textit{Eqn. II. 5}$$

The free energy of a configuration, $G^{\circ}{}_{\alpha}$, depends on the binding of ligand sites and can be written as:

$$G^{\circ}{}_{\alpha} = \sum_{i} \sigma_i G^{\circ}{}_{i} + \sum_{i\neq j} \sigma_i \sigma_j G^{\circ}{}_{ij} + \sum_{i\neq j\neq k} \sigma_i \sigma_j \sigma_k G^{\circ}{}_{ijk} \qquad \textit{Eqn. II. 6}$$

With, i, j and $k \in \{1,2,3,pro\}$.

Here, $G^{\circ}{}_{i}$, $G^{\circ}{}_{ij}$, and $G^{\circ}{}_{ijk}$ are the binding energy of each bound ligand, secondary interaction energies between each pair of bound ligands, and tertiary interaction energies between every three of bound ligands, respectively.

According to *Eqn.II.6*, each single-site binding energy can only contribute to $G^{\circ}{}_{\alpha}$ if the corresponding site is occupied (σ is equal to 1). Moreover, each interaction energy can only contribute to $G^{\circ}{}_{\alpha}$ if the cognate binding sites are all occupied (product of σ's is equal to 1). Notably, we assume the free energy for the configuration with no site being bound ($[\sigma_1 = 0, \sigma_2 = 0, \sigma_3 = 0, \sigma_{pro} = 0]$) to be 0.

To make notations comprehensive for the following sections, below we show how to expand the summation terms in *Eqn. II. 6* as a function of specific energy parameters:

$$\sum_i \sigma_i G^\circ{}_i = \sigma_1 G^\circ{}_1 + \sigma_2 G^\circ{}_2 + \sigma_3 G^\circ{}_3 + \sigma_{pro} G^\circ{}_{pro} \qquad \textit{Eqn. II. 7}$$

$$\sum_{i \neq j} \sigma_i \sigma_j G^\circ{}_{ij} = \sigma_1 \sigma_2 G^\circ{}_{12} + \sigma_1 \sigma_3 G^\circ{}_{13} + \sigma_1 \sigma_{pro} G^\circ{}_{1pro} + \sigma_2 \sigma_3 G^\circ{}_{23} + \sigma_2 \sigma_{pro} G^\circ{}_{2pro} + \sigma_3 \sigma_{pro} G^\circ{}_{3pro} \qquad \textit{Eqn. II. 8}$$

$$\sum_{i \neq j \neq k} \sigma_i \sigma_j \sigma_k G^\circ{}_{ijk} = \sigma_1 \sigma_2 \sigma_3 G^\circ{}_{123} + \sigma_1 \sigma_2 \sigma_{pro} G^\circ{}_{12pro} + \sigma_1 \sigma_3 \sigma_{pro} G^\circ{}_{13pro} + \sigma_2 \sigma_3 \sigma_{pro} G^\circ{}_{23pro} \qquad \textit{Eqn. II. 9}$$

Where $G^\circ{}_1$, $G^\circ{}_2$, $G^\circ{}_3$, and $G^\circ{}_{pro}$ are binding energies of each ligand binding site; $G^\circ{}_{12}$, $G^\circ{}_{13}$, $G^\circ{}_{23}$, and $G^\circ{}_{123}$ are interaction energies between 0A~P bound at each 0A box; $G^\circ{}_{1pro}$, $G^\circ{}_{2pro}$, $G^\circ{}_{3pro}$, $G^\circ{}_{12pro}$, $G^\circ{}_{13pro}$, and $G^\circ{}_{23pro}$ are interaction energies between at least one 0A~P and the RNAP bound at the promoter.

To emphasize, for those configurations where the promoter is bound ($\alpha \in \alpha_T$), the free energy of these configurations ($G^\circ{}_{\alpha \in \alpha_T}$) assumes $\sigma_{pro} = 1$. Conversely, the free energy of all promoter-unbound configurations ($G^\circ{}_{\alpha \in TF}$) assumes $\sigma_{pro} = 0$.

(ii.2) Maximum initiation rate, v_{max}

The maximum transcription rate of configuration *α* can be written as:

$$v_{max_\alpha} = exp\,(-G_\alpha^\dagger) \qquad \textit{Eqn. II. 10}$$

Where, $G_\alpha^\dagger$ is the activation energy for the transcription reaction. $G_\alpha^\dagger$ can be decomposed as:

$$G_\alpha^\dagger = \sum_i \sigma_i G_i^\dagger + \sum_{i \neq j} \sigma_i \sigma_j G_{ij}^\dagger + \sum_{i \neq j \neq k} \sigma_i \sigma_j \sigma_k G_{ijk}^\dagger \qquad \textit{Eqn. II. 11}$$

With, i, j and $k \in \{1,2,3, pro\}$.

Here, $G_i^\dagger$, $G_{ij}^\dagger$, and $G_{ijk}^\dagger$ are the activation energy resulted from each site being bound, activation energy resulted from a pair of sites being bound, and activation energy from three sites being bound, respectively. Further, we assumed that $G_{ij}^\dagger$ and $G_{ijk}^\dagger$ are nonzero.

According to *Eqn. II. 11*, each single-site binding energy can only contribute to $G_{\alpha}^{\dagger}$ if the corresponding site is occupied (σ is equal to 1). Also, each interaction energy can only contribute to $G_{\alpha}^{\dagger}$ if the cognate binding sites are all occupied (product of σ's is equal to 1).

(III) Biophysically model promoter activity for DNA with two promoters

The model framework described above (Supplementary Section II) can be extended to compute gene expression for strains with both P*v* and P*s* promoters present ("P*v*P*s* strain"). For a gene segment with two promoters and three transcription factor binding sties, each possible configuration (α) can be represented as:

$$\alpha = \left[\sigma_1, \sigma_2, \sigma_3, \sigma_{pro}^{v}, \sigma_{pro}^{s}\right] \qquad \textit{Eqn. III. 1}$$

Where, $\sigma_{pro}^{v}, \sigma_{pro}^{s}$ denote P*v* bound/unbound or P*s* bound/unbound, respectively.

In a two-promoter system, transcription is possible if at least one promoter is bound. Therefore, configurations where only P*v* is bound (α_v), configurations where only *Ps* is bound (α_s), and configurations where both P*v* and P*s* are bound (α_{vs}) all contribute to the effective transcription rate. As such the effective transcription rate of a two-promoter mutant strain ($v_{MS_{two\ promoters}}^{effective}$) can be written as:

$$v_{MS_{two\ promoters}}^{effective} = \sum_{\alpha \in \alpha_v} v_{max_\alpha} P_\alpha + \sum_{\alpha \in \alpha_s} v_{max_\alpha} P_\alpha + \sum_{\alpha \in \alpha_{vs}} v_{max_\alpha} P_\alpha \qquad \textit{Eqn. III. 2}$$

Where,

$$\alpha_v = \left[\sigma_1, \sigma_2, \sigma_3, \sigma_{pro}^{v} = 1, \sigma_{pro}^{s} = 0\right] \qquad \textit{Eqn. III. 3}$$

$$\alpha_s = \left[\sigma_1, \sigma_2, \sigma_3, \sigma_{pro}^{v} = 0, \sigma_{pro}^{s} = 1\right] \qquad \textit{Eqn. III. 4}$$

$$\alpha_{vs} = \left[\sigma_1, \sigma_2, \sigma_3, \sigma_{pro}^{v} = 1, \sigma_{pro}^{s} = 1\right] \qquad \textit{Eqn. III. 5}$$

Similarly to *Eqn.II.3,* the probability of RNAP binding to a particular configuration, P_α, is computed using Boltzmann weights of configurations (BW_α) (5–7, 10). This way, one can explicitly express P_α depending on whether the configuration has P*v* bound (α_v), P*s* bound (α_s), or P*v and* P*s* both bound (α_{vs}), as shown below:

$$P_{\alpha\in\alpha_v} = \frac{BW_{\alpha\in\alpha_v}}{\sum BW_\alpha} = \frac{BW_{\alpha\in\alpha_v}}{\sum BW_{\alpha\in\alpha_v} + \sum BW_{\alpha\in\alpha_s} + \sum BW_{\alpha\in\alpha_{vs}} + \sum BW_{\alpha\in\alpha_{TF}}}$$ *Eqn. III. 6*

$$P_{\alpha\in\alpha_s} = \frac{BW_{\alpha\in\alpha_s}}{\sum BW_\alpha} = \frac{BW_{\alpha\in\alpha_s}}{\sum BW_{\alpha\in\alpha_v} + \sum BW_{\alpha\in\alpha_s} + \sum BW_{\alpha\in\alpha_{vs}} + \sum BW_{\alpha\in\alpha_{TF}}}$$ *Eqn. III. 7*

$$P_{\alpha\in\alpha_{vs}} = \frac{BW_{\alpha\in\alpha_{vs}}}{\sum BW_\alpha} = \frac{BW_{\alpha\in\alpha_{vs}}}{\sum BW_{\alpha\in\alpha_v} + \sum BW_{\alpha\in\alpha_s} + \sum BW_{\alpha\in\alpha_{vs}} + \sum BW_{\alpha\in\alpha_{TF}}}$$ *Eqn. III. 8*

Since Boltzmann weights of each configuration depends on the free energy of the configuration, the concentration of the bound ligands, and the cooperativity of the ligands, one can further expand *Eqn.III.6-8* as:

$$P_{\alpha\in\alpha_v} = \frac{exp\left(-G^\circ{}_{\alpha\in\alpha_v}\right) \times [0A{\sim}P]^{N_{0A}} \times [R^v]}{Z}$$ *Eqn. III. 9*

$$P_{\alpha\in\alpha_s} = \frac{exp\left(-G^\circ{}_{\alpha\in\alpha_s}\right) \times [0A{\sim}P]^{N_{0A}} \times [R^s]}{Z}$$ *Eqn. III. 10*

$$P_{\alpha\in\alpha_{vs}} = \frac{exp\left(-G^\circ{}_{\alpha\in\alpha_{vs}}\right) \times [0A{\sim}P]^{N_{0A}} \times [R^v][R^s]}{Z}$$ *Eqn. III. 11*

Where $[R^v]$ and $[R^s]$ are the concentration of RNAP bound at promoter *Pv* and *Ps*, respectively. Meanwhile, *Z*, the statistical weight of all configurations, can be written as:

$$Z = Z_1 + Z_2 + Z_3 + Z_4$$ *Eqn. III. 12*

Where,

$$Z_1 = \sum_{\alpha \in \alpha_v} exp\left(-G^{\circ}{}_{\alpha \in \alpha_v}\right) \times [0A{\sim}P]^{N_{0A}} \times [R^v] \qquad \textit{Eqn. III. 13}$$

$$Z_2 = \sum_{\alpha \in \alpha_s} exp\left(-G^{\circ}{}_{\alpha \in \alpha_s}\right) \times [0A{\sim}P]^{N_{0A}} \times [R^s] \qquad \textit{Eqn. III. 14}$$

$$Z_3 = \sum_{\alpha \in \alpha_{vs}} exp\left(-G^{\circ}{}_{\alpha \in \alpha_{vs}}\right) \times [0A{\sim}P]^{N_{0A}} \times [R^v][R^s] \qquad \textit{Eqn. III. 15}$$

$$Z_4 = \sum_{\alpha \in \alpha_{TF}} exp\left(-G^{\circ}{}_{\alpha \in \alpha_{TF}}\right) \times [0A{\sim}P]^{N_{0A}} \qquad \textit{Eqn. III. 16}$$

Similar to *Eqn. II. 6*, for a two-promoter system, $G^{\circ}{}_{\alpha}$, depends on the binding of ligand sites and can be written as:

$$G^{\circ}{}_{\alpha} = \sum_{i} \sigma_i G^{\circ}{}_i + \sum_{i \neq j} \sigma_i \sigma_j G^{\circ}{}_{ij} + \sum_{i \neq j \neq k} \sigma_i \sigma_j \sigma_k G^{\circ}{}_{ijk} \qquad \textit{Eqn. III. 17}$$

With*,* i, j and $k \in \{1,2,3, pro^v, pro^s\}$

The summation terms here can be further decomposed in specific energy parameters as in *Eqn. II. 7-9*. However, due to the existence of 2^5 (three 0A boxes and two promoters) total possible configurations, the decomposition becomes too extensive to be shown.

Also, the common existing parameters between *Eqn. III.17* and *Eqn. II.6* have the same value (e.g., the interaction energy of 0A1 and 0A2 box, $G^{\circ}{}_{12}$, is assumed to be the same, independently of how many promoters the mutant strain has). Noteworthy, the models do not assume the existence of interaction energy between the two promoters, with or without 0A boxes bound.

Lastly, due to the additivity observed in the empirical data, we assume that, for configurations with the same 0A boxes being occupied, v_{max_α} is additive between the two promoters. As such, *Eqn. III. 2* can be rewritten as:

$$v^{effective}_{MS_{two\ promoters}} = \sum_{\alpha \in \alpha_v} v_{max_\alpha} P_\alpha + \sum_{\alpha \in \alpha_s} v_{max_\alpha} P_\alpha + \sum_{\alpha} (v_{max_{\alpha \in \alpha_v}} + v_{max_{\alpha \in \alpha_s}}) P_{\alpha \in \alpha_{vs}} \qquad \textit{Eqn. III. 18}$$

Where, $v_{max_{\alpha\in\alpha_v}}$ and $v_{max_{\alpha\in\alpha_s}}$ are the maximum transcription initiation rates unique to P*v* and P*s* promoter, respectively. The values of $v_{max_{\alpha\in\alpha_v}}$ and $v_{max_{\alpha\in\alpha_s}}$ are the same as v_{max_α} for each respective single promoter model. Noteworthy, this assumption does not necessarily result in an additivity level equal to 1 for all the biophysical models tested.

We also note that in the main manuscript, for simplicity, Section 2.5 configurations are shown as a 3-digit number. These configurations all assume promoter(s) are bound. That is, notation '101' for 'P*v* strains' in the main manuscript is, in fact, $[\sigma_1 = 1, \sigma_2 = 0, \sigma_3 = 1, \sigma_{pro}^{v} = 1]$. Similarly, the notation '100' for 'P*v*P*s* strains' in the main manuscript is in fact $[\sigma_1 = 1, \sigma_2 = 0, \sigma_3 = 0, \sigma_{pro}^{v} = 1,$ $\sigma_{pro}^{s} = 1]$.

(IV) Biophysical models with different types of transcription control

In the previous section (Supplementary Section II and III), we formulated the biophysical framework to model the effective transcription rate of a gene regulated by a single- or dual-promoter system. We assume TF can affect promoter activity by two different control mechanisms: thermodynamic or kinetic (Supplementary Figure S12 and S13). In the following section, we introduce how each mechanism is quantitively represented. Then, within each type of control, we further describe how it is applied to a single- and dual-promoter system. Noteworthy, since 0A~P is the transcription factor in this case, we use "TF" and "0A~P" interchangeably, and "TF binding site" and "0A box" interchangeably.

(iv.1) Biophysical model with purely thermodynamic control

A purely thermodynamic model assumes TF binding affects the probability of RNAP binding to the promoter but has no impact on the overall transcription rate (Supplementary Figure S12). Below we show how we applied such assumption to the single- and dual-promoter system.

(iv.1.a) For a single-promoter mutant strain

In our study, the single promoter system corresponds to single-promoter mutant strains with either P*v* or P*s* promoter. Since thermodynamic control has no effect on the overall transcription rate, the v_{max_α}, in *Eqn. II.2,* has the same value for all configurations (α).

$$v_{max_{\alpha=0001}} = v_{max_{\alpha=0011}} = \cdots = v_{max_{\alpha=1111}} \qquad \textit{Eqn. IV. 1}$$

Instead, TF binding changes the transcription rate by affecting the probability of RNAP binding, specifically, by assuming nonzero interaction energy between any bound 0A~P and bound RNAP. These include 0A~P/RNAP secondary energies ($G^{\circ}{}_{ij}$, $i \in \{1;2;3\}$, $j \in \{promoter\}$) and 0A~P/0A~P/RNAP tertiary energies ($G^{\circ}{}_{ijk}$, $i \in \{1;2;3\}$, $i \neq j \in \{1;2;3\}$, $k \in \{promoter\}$). The sign of these interaction energies determines the mechanism of action as described below:

$$G^{\circ}{}_{ij} \begin{cases} < 0: activator \\ > 0: repressor \end{cases} \text{where } i \in \{1;2;3\}, j \in \{promoter\} \qquad \textit{Eqn. IV. 2}$$

$$G^{\circ}{}_{ijk} \begin{cases} < 0: activator \\ > 0: repressor \end{cases} \text{where } i \in \{1;2;3\}, i \neq j \in \{1;2;3\}, k \in \{promoter\} \qquad \textit{Eqn. IV. 3}$$

Given that all maximum transcription rates are identical regardless of the configuration, *Eqn. II. 2* for "P*v strains*" and "P*s strains*" can be simplified into:

$$v_{MS_{one\,promoter}}^{effective^{v}} = v_{max}^{v} \sum_{\alpha \in \alpha_T} P_{\alpha} \qquad \textit{Eqn. IV. 4}$$

$$v_{MS_{one\,promoter}}^{effective^{s}} = v_{max}^{s} \sum_{\alpha \in \alpha_T} P_{\alpha} \qquad \textit{Eqn. IV. 5}$$

Where v_{max}^{v} and v_{max}^{s} are maximum overall transcription rates for "P*v strains*" and "P*s strains*", respectively.

(iv.1.b) For a double-promoter mutant strain

In our study, the double-promoter system corresponds to the mutant strains with both P*v* and P*s* promoters, 'P*v*P*s* strains'. Given *Eqn. III. 18* and *Eqn. IV.4-5*, the two-promoter effective transcription rate of a mutant strain with two promoters, under purely thermodynamic control, can be described as:

$$v_{MS_{two\ promoters}}^{effective^{vs}} = v_{max}^{v} \sum_{\alpha \in \alpha_v} P_\alpha + v_{max}^{s} \sum_{\alpha \in \alpha_s} P_\alpha + (v_{max}^{v} + v_{max}^{s}) \sum_{\alpha \in \alpha_{vs}} P_\alpha \qquad \textit{Eqn. IV. 6}$$

Notably, a purely thermodynamic control model is generally not additive (Figure S5), i.e., the activity of the double promoters cannot be estimated by adding together the single promoter's activity. However, under certain parameter regimes, it can be additive. Below, we explore other parameter regimes in which additivity is possible, assuming the simplest model: two promoters and a single 0A box, specifically 0A3.

To compute additivity level, we first compute single- and dual-promoter transcription rate. For a single-promoter strain in accordance with *Eqn. II. 2,* there are two promoter-bound configurations that contribute to the effective transcription rate, $[\sigma_1 = 0, \sigma_2 = 0, \sigma_3 = 0, \sigma_{pro} = 1]$ and $[\sigma_1 = 0, \sigma_2 = 0, \sigma_3 = 1, \sigma_{pro} = 1]$. The effective transcription rate can then be expanded using *Eqn. II. 5-7,* with assumptions of thermodynamic control (Section iv. 1. a):

$$v_{one\ promoter}^{effective^{i}} = v_{max}^{i} \frac{[R^i]\exp\left(-G^{\circ i}_{pro}\right) + [R^i][0A{\sim}P]^{N_{0A}}\exp\left(-G^{\circ i}_{pro} - G^{\circ}{}_{3} - G^{\circ i}_{3pro}\right)}{1 + [0A{\sim}P]^{N_{0A}}\exp(-G^{\circ}{}_{3}) + [R^i]\exp\left(-G^{\circ i}_{pro}\right) + [R^i][0A{\sim}P]^{N_{0A}}\exp\left(-G^{\circ i}_{pro} - G^{\circ}{}_{3} - G^{\circ i}_{3pro}\right)} \qquad \textit{Eqn. IV. 7}$$

Where, i ϵ {s,v} depending if it refers to P*s* or P*v* promoter. The variable $G^{\circ}{}_{pro}$ is used to represent promoter-specific RNAP binding free energy. The variable $G^{\circ}{}_{3pro}$ is the RNAP-TF interaction and v_{max} is the maximum transcription initiation rate. The variable $G^{\circ}{}_{3}$ is the binding affinity of 0A~P at 0A3.

Next, using the dual-promoter modeling framework (*Eqn. III. 9-17*) and assuming thermodynamic control (*Eqn. IV. 6*), we can write the effective transcription rate of the dual-promoter strain (P*v*P*s* strain) as:

$$v_{two\ promoter}^{effective^{vs}} = Vv + Vs + Vvs \qquad \textit{Eqn. IV. 8}$$

where,

$$Vv = v_{max}^{v} \frac{\left([R^v]\exp\left(-G^{\circ v}_{pro}\right) + [R^v][0A{\sim}P]^{N_{0A}}\exp\left(-G^{\circ v}_{pro} - G^{\circ}{}_{3} - G^{\circ v}_{3pro}\right)\right)}{Z} \qquad \textit{Eqn. IV. 9}$$

$$Vs = v_{max}^{s} \frac{\left([R^s]\exp\left(-G^{\circ s}_{pro}\right) + [R^s][0A{\sim}P]^{N_{0A}}\exp\left(-G^{\circ s}_{pro} - G^{\circ}{}_{3} - G^{\circ s}_{3pro}\right)\right)}{Z} \qquad \textit{Eqn. IV. 10}$$

$$Vvs = (v_{max}^{v} + v_{max}^{s}) \frac{\left([R^v][R^s]\exp\left(-G^{\circ v}_{pro} - G^{\circ s}_{pro}\right) + [R^v][R^s][0A{\sim}P]^{N_{0A}}\exp\left(-G^{\circ v}_{pro} - G^{\circ s}_{pro} - G^{\circ}{}_{3} - G^{\circ v}_{3pro} - G^{\circ s}_{3pro}\right)\right)}{Z} \qquad \textit{Eqn. IV. 11}$$

The partition function *Z* is equal to:

$$Z = Z_a + Z_b + Z_c \qquad \textit{Eqn. IV. 12}$$

where,

$$Z_a = 1 + [0A{\sim}P]^{N_{0A}} \exp(-G^{\circ}{}_3) + [R^v] \exp\left(-G^{\circ v}_{pro}\right) + [R^s] \exp\left(-G^{\circ s}_{pro}\right) \qquad \textit{Eqn. IV. 13}$$

$$Z_b = [R^v][0A{\sim}P]^{N_{0A}} \exp\left(-G^{\circ v}_{pro} - G^{\circ}{}_3 - G^{\circ v}_{3pro}\right) + [R^s][0A{\sim}P]^{N_{0A}} \exp\left(-G^{\circ s}_{pro} - G^{\circ}{}_3 - G^{\circ s}_{3pro}\right) \qquad \textit{Eqn. IV. 14}$$

$$Z_c = [R^v][R^s] \exp\left(-G^{\circ v}_{pro} - G^{\circ s}_{pro}\right) + [R^v][R^s][0A{\sim}P]^{N_{0A}} \exp\left(-G^{\circ v}_{pro} - G^{\circ s}_{pro} - G^{\circ}{}_3 - G^{\circ v}_{3pro} - G^{\circ s}_{3pro}\right) \qquad \textit{Eqn. IV. 15}$$

Next, we simplify the above expressions by defining:

$$[0A{\sim}P]^{N_{0A}} \exp(-G^{\circ}{}_3) = [0A{\sim}P]_{norm} \qquad \textit{Eqn. IV. 16}$$

$$[R^i] \exp\left(-G^{\circ i}_{pro}\right) = [R^i]_{norm} \qquad \textit{Eqn. IV. 17}$$

$$\exp\left(-G^{\circ i}_{3pro}\right) = \varphi_{3i} \qquad \textit{Eqn. IV. 18}$$

Where, i ϵ {s,v}

Consequently, single-promoter transcription rate in *Eqn. IV. 7* can be written as:

$$v_{3_{one\ promoter}}^{effective^i} = v^i_{max} \frac{[R^i]_{norm}\left(1 + \varphi_{3i}[0A{\sim}P]_{norm}\right)}{1 + [0A{\sim}P]_{norm} + [R^i]_{norm}\left(1 + \varphi_{3i}[0A{\sim}P]_{norm}\right)} \qquad \textit{Eqn. IV. 19}$$

And dual-promoter transcription rate in *Eqn. IV. 8-11* can be written as:

$$v_{3_{two\ promoter}}^{effective^{vs}} = Vv + Vs + Vvs \qquad \textit{Eqn. IV. 20}$$

where,

$$Vv = v^v_{max} \frac{[R^v]_{norm}(1 + \varphi_{3v}[0A{\sim}P]_{norm})}{Z} \qquad \textit{Eqn. IV. 21}$$

$$Vs = v^s_{max} \frac{[R^s]_{norm}(1 + \varphi_{3s}[0A{\sim}P]_{norm})}{Z} \qquad \textit{Eqn. IV. 22}$$

$$Vvs = (v^v_{max} + v^s_{max}) \frac{[R^v]_{norm}[R^s]_{norm}(1 + \varphi_{3v}\varphi_{3s}[0A{\sim}P]_{norm})}{Z} \qquad \textit{Eqn. IV. 23}$$

The partition function Z in *Eqn. IV. 12-15* is rewritten as

$$Z = Z_c + Z_d \qquad \textit{Eqn. IV. 24}$$

Where,

$$Z_c = 1 + [0A{\sim}P]_{norm} + [R^v]_{norm}(1 + \varphi_{3v}[0A{\sim}P]_{norm}) + [R^s]_{norm}(1 + \varphi_{3s}[0A{\sim}P]_{norm}) \qquad \textit{Eqn. IV. 25}$$

$$Z_d = [R^v]_{norm}[R^s]_{norm}(1 + \varphi_{3v}\varphi_{3s}[0A{\sim}P]_{norm}) \qquad \textit{Eqn. IV. 26}$$

From *Eqn. IV. 19*, one can observe that if $[0A{\sim}P]_{norm} \gg 0$ (i.e. reaching saturation), the term "1" and $[R^i]_{norm}$ become negligible and the single-promoter transcription rate can be written as:

$$v_{3_{one\,promoter}}^{effective^i} \approx v_{max}^i \frac{\varphi_{3i}[R^i]_{norm}}{1 + \varphi_{3i}[R^i]_{norm}} \qquad \textit{Eqn. IV. 27}$$

Similarly, for the dual-promoter transcription rate, the term "1", $[R^v]_{norm}$, and $[R^s]_{norm}$ in *Eqn. IV. 20-26* also become negligible. As such, *Eqn. IV. 20* becomes:

$$v_{3_{two\,promoter}}^{effective^{vs}} \approx \frac{v_{max}^v\varphi_{3v}[R^v]_{norm}(1 + \varphi_{3s}[R^s]_{norm}) + v_{max}^s\varphi_{3s}[R^s]_{norm}(1 + \varphi_{3v}[R^v]_{norm})}{1 + \varphi_{3v}[R^v]_{norm} + \varphi_{3s}[R^s]_{norm} + \varphi_{3v}\varphi_{3s}[R^v]_{norm}[R^s]_{norm}} \qquad \textit{Eqn. IV. 28}$$

Comparing *Eqn. IV. 28* to *Eqn. IV. 27*, we observe that *Eqn. IV. 28* corresponds to the sum of the single promoters' equation (*Eqn. IV. 27 for i=s and i=v)*. This suggests that under the regime of TF saturation ($[0A{\sim}P]_{norm} \gg 0$) the thermodynamic control model is additive.

In addition to the regime where TF is saturated, there are other trivial regimes for which a thermodynamical model is additive. For example, if $[R^v]_{norm} \ll 1$ and $[R^s]_{norm} \ll 1$. Due to very weak RNAP binding affinity, the binding probability of RNAP is very small. Alternatively, for $\varphi_{1s} \ll 1$ and $\varphi_{1v} \ll 1$. Due to strong repulsion between RNAP and TF, binding probability of both RNAP approaches 0. In these cases, since transcription rate coming from all promoters is close to zero, transcription rate tends to be additive.

(iv.2) Biophysical model with purely kinetic control

A purely kinetic model assumes TF binding affects the overall transcription rate but has no impact on the recruitment of RNAP to the promoter (Supplementary Figure S13). Below we show how we applied such assumption to the single- and dual-promoter system model framework.

(iv.2.a) For a single-promoter mutant strain

In the kinetic control mechanism, the RNAP binding probability is not affected by 0A~P binding. As such, there is no interaction between RNAP and 0A~P. Instead, for kinetic control, the effect

of TF binding is on the maximum transcription rate. As such, v_{max_α} in *Eqn. II. 2* is assumed to be different for 0A~P bound and 0A~P unbound configurations.

In addition, the relative magnitude of v_{max_α} reflects the mechanism of action. If v_{max_α} of a 0A~P bound configuration is smaller than the 0A~P unbound configuration, the binding of the 0A box has a repressive effect, or vice versa, as described below:

$$v_{max_{\alpha_T}} \begin{cases} < v_{max_{\alpha=000}}: \text{repressor} \\ > v_{max_{\alpha=000}}: \text{activator} \end{cases}$$ *Eqn. IV. 29*

Where, $\alpha_T = [\sigma_1, \sigma_2, \sigma_3, \sigma_{promoter} = 1]$.

The decomposition of the effective transcription rate *(Eqn. II.2*), for the kinetic control model, is described in the following paragraphs.

First, given that secondary (G°_{ij}) and tertiary interaction (G°_{ijk}) energies between bound 0A~P and bound RNAP are null (zero) in a kinetic model, *Eqn. II.6* can be written as follows for promoter-bound (α_T, where $\sigma_{pro} = 1$) and promoter-unbound (α_{TF}, where $\sigma_{pro} = 0$) configurations, in accordance with *Eqn. II. 7-9*:

$$G^{\circ}_{\alpha\in\alpha_T} = \sigma_1 G^{\circ}_1 + \sigma_2 G^{\circ}_2 + \sigma_3 G^{\circ}_3 + G^{\circ}_{pro} + \sigma_1\sigma_2 G^{\circ}_{12} + \sigma_1\sigma_3 G^{\circ}_{13} + \sigma_2\sigma_3 G^{\circ}_{23} + \sigma_1\sigma_2\sigma_3 G^{\circ}_{123}$$ *Eqn. IV. 30*

$$G^{\circ}_{\alpha\in\alpha_{TF}} = \sigma_1 G^{\circ}_1 + \sigma_2 G^{\circ}_2 + \sigma_3 G^{\circ}_3 + \sigma_1\sigma_2 G^{\circ}_{12} + \sigma_1\sigma_3 G^{\circ}_{13} + \sigma_2\sigma_3 G^{\circ}_{23} + \sigma_1\sigma_2\sigma_3 G^{\circ}_{123}$$ *Eqn. IV. 31*

Note that *Eqn. IV.8* can also be written as a function of *Eqn. IV.9*:

$$G^{\circ}_{\alpha\in\alpha_T} = G^{\circ}_{\alpha\in\alpha_{TF}} + G^{\circ}_{pro}$$ *Eqn. IV. 32*

As such, the binding probability of RNAP to the promoter, *Eqn. II.5,* becomes:

$$P_\alpha = \frac{exp(-G^{\circ}_{pro}) \times [R]}{1 + exp(-G^{\circ}_{pro}) \times [R]} \times \frac{exp(-G^{\circ}_{\alpha\in\alpha_{TF}}) \times [0A{\sim}P]^{N_{0A}}}{\sum_{\alpha\in\alpha_{TF}} exp(-G^{\circ}_{\alpha\in\alpha_{TF}})\,[0A{\sim}P]^{N_{0A}}}$$ *Eqn. IV. 33*

Note that the factorization of the term $\frac{exp(-G^{\circ}{}_{pro})\times[R]}{1+exp(-G^{\circ}{}_{pro})\times[R]}$ suggests the effect of RNAP is independent from the effect of transcription factor on binding probability, as expected in a purely kinetic model.

Finally, the effective transcription rate of a mutant strain ($\boldsymbol{v_{MS}^{effective}}$), *Eqn. II.2,* can be written as:

$$v_{MS_{one\ promoter}}^{effective} = \frac{exp(-G^{\circ}{}_{pro}) \times [R]}{1 + exp(-G^{\circ}{}_{pro}) \times [R]} \sum_{\alpha} v_{max_{\alpha\in\alpha_T}} \times P_{\alpha\in\alpha_{TF}} \qquad \textit{Eqn. IV. 34}$$

Where, the rate can be computed by summing the binding probability of 0A~P ($\alpha \in \alpha_{TF}$), weighted by their corresponding $v_{max_{\alpha\in\alpha_T}}$ (for example, probability of the configuration [$\sigma_1 = 1, \sigma_2 = 0, \sigma_3 = 1$] will be multiplied by the v_{max} of configuration [$\sigma_1 = 1, \sigma_2 = 0, \sigma_3 = 1, \sigma_{pro}^{v} = 1$]), and multiplied by the factorization term.

(iv.2.b) For a double-promoter mutant strain

Below we show how to mathematically formulate the effective transcription rate of a dual-promoter system assuming TF effects are kinetic. We also show analytically that a purely kinetic control model leads to an additive transcription rate between two promoters.

First, in the dual-promoter system, the free energy for configurations with no promoter bound ($G^{\circ}{}_{\alpha\in\alpha_{TF}}$) are equal to that in the single-promoter system (*Eqn. IV.9*). Further, since $G^{\circ}{}_{\alpha\in\alpha_{TF}}$ does not account with interactions between 0A~P and RNAP bound to either promoter, and given there is no interaction between the two bound promoters, the energy for each group of configurations, $G^{\circ}{}_{\alpha\in\alpha_v}$, $G^{\circ}{}_{\alpha\in\alpha_s}$, and $G^{\circ}{}_{\alpha\in\alpha_{vs}}$, in *Eqn. III.9-11,* can be conveniently expressed as a function of $G^{\circ}{}_{\alpha\in\alpha_{TF}}$.

$$G^{\circ}{}_{\alpha\in\alpha_v} = G^{\circ}{}_{\alpha\in\alpha_{TF}} + G^{\circ v}_{pro} \qquad \textit{Eqn. IV. 35}$$

$$G^{\circ}{}_{\alpha\in\alpha_s} = G^{\circ}{}_{\alpha\in\alpha_{TF}} + G^{\circ s}_{pro} \qquad \textit{Eqn. IV. 36}$$

$$G^{\circ}{}_{\alpha\in\alpha_{vs}} = G^{\circ}{}_{\alpha\in\alpha_{TF}} + G^{\circ v}_{pro} + G^{\circ s}_{pro} \qquad \textit{Eqn. IV. 37}$$

With this simplification, the binding probabilities in *Eqn. III. 9-11* can be written as:

$$P_{\alpha\in\alpha_v} = \frac{exp(-G^{\circ}{}_{\alpha\in\alpha_v}) \times [0A\sim P]^{N_{0A}} \times [R^v]}{Z} = \frac{exp(-G^{\circ v}_{pro}) \times [R^v] \times exp(-G^{\circ}{}_{\alpha\in\alpha_{TF}}) \times [0A\sim P]^{N_{0A}}}{Z} \quad \textit{Eqn. IV. 38}$$

$$P_{\alpha\in\alpha_s} = \frac{exp(-G^{\circ}{}_{\alpha\in\alpha_s}) \times [0A\sim P]^{N_{0A}} \times [R^s]}{Z} = \frac{exp(-G^{\circ s}_{pro}) \times [R^s] \times exp(-G^{\circ}{}_{\alpha\in\alpha_{TF}}) \times [0A\sim P]^{N_{0A}}}{Z} \quad \textit{Eqn. IV. 39}$$

$$P_{\alpha\in\alpha_{vs}} = \frac{exp(-G^{\circ}{}_{\alpha\in\alpha_{vs}}) \times [0A\sim P]^{N_{0A}} \times [R^v][R^s]}{Z} = \frac{exp(-G^{\circ v}_{pro} - G^{\circ s}_{pro}) \times [R^v] \times [R^s] \times exp(-G^{\circ}{}_{\alpha\in\alpha_{TF}}) \times [0A\sim P]^{N_{0A}}}{Z} \quad \textit{Eqn. IV. 40}$$

Given *Eqn. IV.13-15, Z,* the statistical weight of all configurations, can be written as:

$$Z = \underbrace{(1 + exp(-G^{\circ v}_{pro}) \times [R^v])}_{Pv\ unbound/bound} \underbrace{(1 + exp(-G^{\circ s}_{pro}) \times [R^s])}_{Ps\ unbound/bound} \underbrace{(1 + \sum_{\alpha\in\alpha_{TF}} exp(-G^{\circ}{}_{\alpha\in\alpha_{TF}}) \times [0A\sim P]^{N_{0A}})}_{TF\ unbound/bound} \quad \textit{Eqn. IV. 41}$$

In accordance with *Eqn.III.2,* the effective transcription rate ($v^{effective}_{MS_{two\ promoters}}$) is then each promoter-specific configuration weighted by its promoter-specific v_{max_α}:

$$v^{effective}_{MS_{two\ promoters}} = v1 + v2 + v3 \quad \textit{Eqn. IV. 42}$$

Where, $v1$, $v2$, and $v3$ can be written as:

$$v1 = \frac{exp(-G^{\circ v}_{pro})[R^v] \sum_\alpha v_{max_{\alpha\in\alpha_v}} \times P_{\alpha\in\alpha_{TF}}}{(1 + exp(-G^{\circ v}_{pro}) \times [R^v])(1 + exp(-G^{\circ s}_{pro}) \times [R^s])} \quad \textit{Eqn. IV. 43}$$

$$v2 = \frac{exp(-G^{\circ s}_{pro})[R^s] \sum_\alpha v_{max_{\alpha\in\alpha_s}} \times P_{\alpha\in\alpha_{TF}}}{(1 + exp(-G^{\circ v}_{pro}) \times [R^v])(1 + exp(-G^{\circ s}_{pro}) \times [R^s])} \quad \textit{Eqn. IV. 44}$$

$$v3 = \frac{exp(-G^{\circ v}_{pro} - G^{\circ s}_{pro})[R^v][R^s] \sum_\alpha \left(v_{max_{\alpha\in\alpha_v}} + v_{max_{\alpha\in\alpha_s}}\right) \times P_{\alpha\in\alpha_{TF}}}{(1 + exp(-G^{\circ v}_{pro}) \times [R^v])(1 + exp(-G^{\circ s}_{pro}) \times [R^s])} \quad \textit{Eqn. IV. 45}$$

Here, $\alpha_{T_{TF}}$ are the set of configurations concerning the binding of TF only. The variables $v_{max_{\alpha\in\alpha_v}}$ and $v_{max_{\alpha\in\alpha_s}}$ are the maximum transcription rate for each promoter-specific configuration when promoter is bound.

Further, one can expand the numerator of $v3$ (*Eqn. IV.23)* into:

$$v3 = \frac{exp\left(-G^{\circ v}_{pro} - G^{\circ s}_{pro}\right)[R^v][R^s]\left(\sum_\alpha v_{max_{\alpha\in\alpha_v}} \times P_{\alpha\in\alpha_{TF}} + \sum_\alpha v_{max_{\alpha\in\alpha_s}} \times P_{\alpha\in\alpha_{TF}}\right)}{(1 + exp\left(-G^{\circ v}_{pro}\right) \times [R^v])(1 + exp\left(-G^{\circ s}_{pro}\right) \times [R^s])} \qquad \textit{Eqn. IV. 46}$$

As such, now *Eqn. IV.20* be rearranged into

$$v^{effective}_{MS_{two\ promoters}} = \frac{exp\left(-G^{\circ v}_{pro}\right)[R^v]\sum_\alpha v_{max_{\alpha\in\alpha_v}} \times P_{\alpha\in\alpha_{TF}}}{(1 + exp\left(-G^{\circ v}_{pro}\right) \times [R^v])} + \frac{exp\left(-G^{\circ s}_{pro}\right)[R^s]\sum_\alpha v_{max_{\alpha\in\alpha_s}} \times P_{\alpha\in\alpha_{TF}}}{(1 + exp\left(-G^{\circ s}_{pro}\right) \times [R^s])} \qquad \textit{Eqn. IV. 47}$$

Which is exactly the sum of single-promoter effective transcription rate (*Eqn. IV.12*) between two promoters. This additivity property of the purely kinetic model is in agreement with the additivity observed in the experimental data.

(iv.3) Model with mixed control

The types of transcriptional control described in Supplementary Section iv.1 and iv.2 assume 0A~P acts on both promoters with the same type of control: thermodynamically or kinetically. It is also possible that transcriptional regulation at each promoter is unique, including two possibilities: i) kinetic P*v* and thermodynamically regulated P*s*; ii) thermodynamic P*v* and kinetically regulated P*s*. Below we introduce the model framework of single- and double-promoter models with mixed control, assuming the case of kinetic P*v* and thermodynamically controlled P*s*.

For simplicity, the framework for single-promoter thermodynamic model and for single-promoter kinetic model have been developed in Supplementary Section iv.1.a and Supplementary Section iv.2.a (*Eqn. IV. 5* and *Eqn. IV.12*) and will not be detailed here.

To compute the effective transcription rate of a dual-promoter mixed model, we follow the general framework introduced in Supplementary Section III to calculate the RNAP binding probability. Using *Eqn. III.6* (generalized form of the Boltzmann factor for P*v* bound configurations), the contribution from P*v* promoter due to kinetic control can be written using the same rationale as in Supplementary Section iv.2.b*:*

$$P_{\alpha\in\alpha_v} = \frac{exp\left(-G^{\circ v}_{pro}\right) \times [R^v] \times exp\left(-G^{\circ}{}_{\alpha\in\alpha_{TF}}\right) \times [0A{\sim}P]^{N_{0A}}}{\sum_\alpha exp\left(-G^{\circ}{}_{\alpha}\right) \times [0A{\sim}P]^{N_{0A}} \times [R^v] \times [R^s]} \qquad \textit{Eqn. IV. 48}$$

We then assign a maximum transcription initiation rate for all configurations in the two-promoter system. Based on Supplementary Section iv.1, because P*s* is thermodynamically regulated, all v_{max_α} for $\alpha \in \alpha_s$ are the same (here denominated as universal v_{max}^{s}). Based on Supplementary Section iv.2, the kinetically-regulated P*v* will have a unique v_{max_α} for each configuration. In accordance with *Eqn. III. 18*, the effective transcription rate for a dual promoter mutant strain can be written as:

$$v_{MS_{two\ promoters}}^{effective^{sv}} = \frac{v_{max}^{s} \sum_{\alpha \in \alpha_s} P_\alpha + exp\left(-G^{\circ v}_{pro}\right)[R^v] \sum_\alpha v_{max_{\alpha \in \alpha_v}} \times P_{\alpha \in \alpha_{TF}} + \sum_\alpha \left(v_{max_{\alpha \in \alpha_v}} + v_{max}^{s}\right) P_{\alpha \in \alpha_{vs}}}{\sum_\alpha exp(-G^\circ{}_\alpha) \times [0A{\sim}P]^{N_{0A}} \times [R^v] \times [R^s]} \qquad \textit{Eqn. IV. 49}$$

(V) Constrain the biophysical gene regulatory model with EMSA predicted parameters

In this section, we show how parameters predicted from EMSA data are used to constrain the biophysical gene regulatory model.

In Supplementary Section I, we obtained binding dissociation constant for each 0A box ($K_{half_1}, K_{half_2}, K_{half_3}$) and N. We first converted optimized K_{half} parameters into binding energy parameters according to *Eqn. I. 7*, and call these $G_{EMSA_1}, G_{EMSA_2}, G_{EMSA_3}$. Then, we let the binding energy of single 0A boxes ($G^\circ{}_1$, $G^\circ{}_2$, $G^\circ{}_3$, see *Eqn. II. 7*) in the biophysical model equal to $G_{EMSA_1}, G_{EMSA_2}, G_{EMSA_3}$, respectively. In other words, the occupancy of 0A boxes during transcription is assumed to be the same as the occupancy of 0A boxes during EMSA.

However, directly using $G_{EMSA_1}, G_{EMSA_2}, G_{EMSA_3}$ in the biophysical model fails to yield a unique fit (model was found to be degenerate), suggesting the model needs to be more tightly constrained. Interestingly, $G^\circ{}_1$, $G^\circ{}_2$, $G^\circ{}_3$ are consistently found to be more negative than estimated $G_{EMSA_1}, G_{EMSA_2}, G_{EMSA_3}$. As such, to better constrain the model, we assume that there is a common ratio between 0 and 1, α, between K_{EMSA} (half saturation constant estimated from EMSA) and K° (biophysical model half saturation constant) such that:

$$\frac{K^\circ{}_{half_1}}{K_{half_{EMSA1}}} = \frac{K^\circ{}_{half_2}}{K_{half_{EMSA2}}} = \frac{K^\circ{}_{half_3}}{K_{half_{EMSA3}}} = \alpha \qquad \textit{Eqn. V. 1}$$

Where,

$$K^{\circ}_{half_i} = \exp\left(\frac{G^{\circ}_i}{N}\right) \text{ and } K_{half_{EMSAi}} = \exp\left(\frac{G_{EMSAi}}{N}\right) \qquad \textit{Eqn. V. 2}$$

Here, *N* refers to the cooperativity of 0A~P and is predicted to be 4 in accordance with Section I, and the subscript {1,2,3} denotes each 0A box. Given that G_{EMSA} values are known, the degree of freedom in the binding affinity parameter space is decreased from three to one. Under this regime, a unique model solution is found (Table S4).

One physical interpretation for α is that it represents the active fraction of the total 0A protein added to the EMSA. Notably, during gel shift experiment, while the amount of total purified [0A] is explicitly reported, the amount of active form [0A~P] that binds to the DNA fragment is unknown. As a result, the estimated binding affinities of 0A boxes based on reported [0A], i.e., $G_{EMSA_1}, G_{EMSA_2}, G_{EMSA_3}$ from Supplementary Section I, must be adjusted. In other words, the “true” 0A box binding affinities are functions of G_{EMSA} and α.

The model fitting results yields that $\alpha = 0.2$.

(VI) Compute 0A box occupancy probability using best-fit kinetic model parameters

In Result Section 2.4, we showed that the effect of 0A~P binding is saturated at t = 2 h. Here, we first demonstrate how to compute the probability of 0A~P binding to 0A boxes. Then, we show why saturation in 0A box binding probability leads to no change in effective transcription rate as a function of 0A~P concentrations.

The occupancy probability of 0A boxes by 0A~P for each strain is calculated in the same way as described in Section I, *Eqn. I. 6*, *Eqn. I. 8*, and *Eqn. I. 9* for DNA fragments with one, two, and three boxes, respectively, using the best-fit binding energy and interaction energy parameters shown in Supplementary Table S4. Occupancy probability for each mutant strain is shown in Supplementary Figure S6.

Notably, the expressions representing the Boltzmann weights of different 0A~P bound configurations in *Eqn. I. 6*, *Eqn. I. 8*, and *Eqn. I. 9* also appear in the calculation of the Boltzmann weight in *Eqn. II. 4,* in both promoter-bound and promoter-unbound configurations. If there is no time-dependent change in RNAP concentration, and given that the maximum transcription

initiation rate is constant over all time points, there is no change in effective transcription rate in accordance with *Eqn. II. 2*, i.e., saturated 0A~P binding probability will result in a saturated effective transcription rate.

(VII) Bayesian Information Criterion for differentially constrained models

To verify that the model is not overfitted upon the addition of interaction energy parameters, we computed the Bayesian Information Criterion (11) (BIC) for the models with none or up to three secondary interaction energies (G_{12}, G_{13}, G_{23}) being zero.

To compute the BIC, we use the following formula (11):

$$BIC = \log(E) + \frac{N_{pars}}{N_{obs}} \times \log(N_{obs}) \qquad \textit{Eqn. VII. 1}$$

Where *E* is the error of the objective function employed during optimization of the biophysical model (Methods and Section 4.5). The variable N_{pars} is the number of unknown fitting parameters, and N_{obs} is the total number of experimental data points.

The lowest BIC is found in the model with all secondary interaction energies being nonzero (Supplementary Figure Table S5). As such, the model with the existence of higher-order interaction energies does not overfit the experimental data, and the kinetic control model requires the attractions between bound 0A boxes to explain *spo0A* autoregulation.

(VIII) Model-predicted regulatory role of 0A boxes determined from v_{max} and $v^{effective}$

In Figure 6, we examined the model-predicted effect of 0A~P binding at different boxes in two ways: 1) by analyzing the change in the maximum transcription initiation rate of each configuration (v_{max_α}, Figure 6A-C) relative to the configuration '000'; 2) by analyzing the effective transcription rate at t = 9 h of each mutant strain ($v^{effective}_{MS_{one\ promoter}}$ and $v^{effective}_{MS_{two\ promoter}}$, Figure 6D) relative to the 'none' strain. Below, we show that these two tests are analytically equivalent for a single-promoter kinetic control model. We then show that, for the double-promoter kinetic control model, the two

tests are not equivalent, as the 0A~P binding effect on the dual-promoter effective transcription rate is the weighted sum of 0A~P binding effects on P*v* and P*s*, respectively.

(viii.1) for single-promoter strains

Here, we will demonstrate that the relative 0A box effects on v_{max} and $v^{effective}$ are quantitatively equivalent in single-promoter strains. We assume the most complex case with all 0A boxes being present ('123 strain'). Due to the nature of the kinetic control model, in accordance with *Eqn. IV. 12,* the effective transcription rate can be written as a product of the factorization term (denoting RNAP binding) and the sum of 0A~P binding weighted by configuration-dependent maximum transcription initiation rate:

$$v_{123_{one\,promoter}}^{effective^v} = \frac{exp(-G^{\circ v}_{pro})\times[R^v]}{1+exp(-G^{\circ v}_{pro})\times[R^v]}(v^v_{max_{0001}}P_{0000} + v^v_{max_{0011}}P_{0010} + \cdots + v^v_{max_{1111}}P_{1110}) \qquad \textit{Eqn. VIII. 1}$$

$$v_{123_{one\,promoter}}^{effective^s} = \frac{exp(-G^{\circ s}_{pro})\times[R^s]}{1+exp(-G^{\circ s}_{pro})\times[R^s]}(v^s_{max_{0001}}P_{0000} + v^s_{max_{0011}}P_{0010} + \cdots + v^s_{max_{1111}}P_{1110}) \qquad \textit{Eqn. VIII. 2}$$

Here, superscript 'v' or 's' are promoter-specific parameters. The binary subscripts for v_{max} are written in terms of promoter-configurations α_T; the binary subscript for P are written in terms of promoter-unbound configurations α_{TF}, in accordance with Section iv.2.a and *Eqn. IV. 12*. For example, '0011' denotes 0A3 box bound and promoter bound.

At t = 9 h, for strain '123', the probability of all boxes being bound (P_{1110}) is equal to 1 (Figure S6). As such, all other possible configurations have a probability equal to zero. Given this, the effective transcription rate for P*v* and P*s* '123' can be simplified into:

$$v_{123_{one\,promoter}}^{effective^v} = \frac{exp(-G^{\circ v}_{pro})\times[R^v]}{1+exp(-G^{\circ v}_{pro})\times[R^v]}v^v_{max_{1111}}P_{1110} = \frac{exp(-G^{\circ v}_{pro})\times[R^v]}{1+exp(-G^{\circ v}_{pro})\times[R^v]}v^v_{max_{1111}} \qquad \textit{Eqn. VIII. 3}$$

$$v_{123_{one\,promoter}}^{effective^s} = \frac{exp(-G^{\circ s}_{pro})\times[R^s]}{1+exp(-G^{\circ s}_{pro})\times[R^s]}v^s_{max_{1111}}P_{1110} = \frac{exp(-G^{\circ s}_{pro})\times[R^s]}{1+exp(-G^{\circ s}_{pro})\times[R^s]}v^s_{max_{1111}} \qquad \textit{Eqn. VIII. 4}$$

In Figure 6D, the effective transcription rate of each strain is relative to the 'none' strain. Using *Eqn. II. 2, Eqn. II. 5* and *Eqn. II. 6*, the effective transcription rate for 'none' strain can be written as below, with $[0A{\sim}P] = 0$ and $\alpha_T = [0,0,0,1]$:

$$v_{none_{one\ promoter}}^{effective^{v}} = \frac{exp\left(-G^{\circ v}_{pro}\right) \times [R^{v}]}{1 + exp\left(-G^{\circ v}_{pro}\right) \times [R^{v}]} v^{v}_{max_{0001}} \quad \textit{Eqn. VIII. 5}$$

$$v_{none_{one\ promoter}}^{effective^{s}} = \frac{exp\left(-G^{\circ s}_{pro}\right) \times [R^{s}]}{1 + exp\left(-G^{\circ s}_{pro}\right) \times [R^{s}]} v^{s}_{max_{0001}} \quad \textit{Eqn. VIII. 6}$$

The fold change in ‘123’ strain compared to ‘none’ strain, therefore, is the ratio between *Eqn. VIII. 3 and Eqn. VIII. 5*, and the ratio between *Eqn. VIII. 4 and Eqn. VIII. 6*, for P*v* and P*s,* respectively:

$$\frac{v_{123_{one\ promoter}}^{effective^{v}}}{v_{none_{one\ promoter}}^{effective^{v}}} = \frac{v^{v}_{max_{1111}}}{v^{v}_{max_{0001}}} \quad \textit{Eqn. VIII. 7}$$

$$\frac{v_{123_{one\ promoter}}^{effective^{s}}}{v_{none_{one\ promoter}}^{effective^{s}}} = \frac{v^{s}_{max_{1111}}}{v^{s}_{max_{0001}}} \quad \textit{Eqn. VIII. 8}$$

Here, the fold change in effective transcription rate between ‘123’ and ‘none’ strain is the same as normalizing v_{max} of configuration ‘111’ (where maximum number of allowable boxes are bound in ‘123’) by the v_{max} of configuration ‘000’. This is true for all other mutant strains, because of the saturated effect of 0A~P binding at t = 9h for all mutant strains. Moreover, the fold change does not depend on the concentration of RNAP.

(viii.2) for double-promoter strains

Using the additive property of the kinetic control model (*Eqn. IV. 25*) and knowing that 0A~P binding probability is contributed exclusively by a single configuration, which saturates at the last time point (as shown in section viii.1), the effective transcription rate for ‘123’ and ‘none’ for ‘P*v*P*s* strains’ can be directly represented using *Eqn. VIII. 3-6*:

$$v_{123_{two\ promoters}}^{effective^{vs}} = v_{123_{one\ promoter}}^{effective^{v}} + v_{123_{one\ promoter}}^{effective^{s}} = \frac{exp\left(-G^{\circ v}_{pro}\right)\times[R^{v}]}{1+exp\left(-G^{\circ v}_{pro}\right)\times[R^{v}]} v^{v}_{max_{1111}} + \frac{exp\left(-G^{\circ s}_{pro}\right)\times[R^{s}]}{1+exp\left(-G^{\circ s}_{pro}\right)\times[R^{s}]} v^{s}_{max_{1111}} \quad \textit{Eqn. VIII. 9}$$

$$v_{none_{two\ promoters}}^{effective^{vs}} = v_{none_{one\ promoter}}^{effective^{v}} + v_{none_{one\ promoter}}^{effective^{s}} = \frac{exp(-G^{\circ v}_{pro}) \times [R^v]}{1+exp(-G^{\circ v}_{pro}) \times [R^v]} v_{max_{0001}}^{v} + \frac{exp(-G^{\circ s}_{pro}) \times [R^s]}{1+exp(-G^{\circ s}_{pro}) \times [R^s]} v_{max_{0001}}^{s} \quad \text{Eqn. VIII. 10}$$

For convenience, we can define

$$\widetilde{R^i} = \frac{exp(-G^{\circ i}_{pro}) \times [R^i]}{1 + exp(-G^{\circ i}_{pro}) \times [R^i]}$$

with i ∈ $\{s, v\}$.

When computing the ratio between '123' and 'none' for 'P*v*P*s* strains', we obtain the following expression:

$$\frac{v_{123_{two\ promoters}}^{effective^{vs}}}{v_{none_{two\ promoters}}^{effective^{vs}}} = \frac{\widetilde{R^v} v_{max_{1111}}^{v} + \widetilde{R^s} v_{max_{1111}}^{s}}{\widetilde{R^v} v_{max_{0001}}^{v} + \widetilde{R^s} v_{max_{0001}}^{s}} \quad \text{Eqn. VIII. 11}$$

To further simplify this equation, we divide the top and bottom of *Eqn. VIII. 11* by $v_{max_{0001}}^{v} v_{max_{0001}}^{s} \widetilde{R^v} \widetilde{R^s}$. In doing so, we obtain:

$$\frac{v_{123_{two\ promoters}}^{effective^{vs}}}{v_{none_{two\ promoters}}^{effective^{vs}}} = \left(\frac{\frac{1}{v_{max_{0001}}^{s} \widetilde{R^s}}}{\frac{1}{v_{max_{0001}}^{s} \widetilde{R^s}} + \frac{1}{v_{max_{0001}}^{v} \widetilde{R^v}}} \right) \frac{v_{max_{1111}}^{v}}{v_{max_{0001}}^{v}} + \left(\frac{\frac{1}{v_{max_{0001}}^{v} \widetilde{R^v}}}{\frac{1}{v_{max_{0001}}^{s} \widetilde{R^s}} + \frac{1}{v_{max_{0001}}^{v} \widetilde{R^v}}} \right) \frac{v_{max_{1111}}^{s}}{v_{max_{0001}}^{s}} \quad \text{Eqn. VIII. 12}$$

Here, we observe that the effect of 0A~P binding on a two-promoter strain is the weighted sum of the effect on the single-promoter expression, i.e., the weighted sum of the ratio between P*v*-bound and P*s*-bound v_{max} (weighted sum of *Eqn. VIII. 7-8*). In addition, the fold change of the '123' strain in relative to 'none' strain now depends on the concentration of RNAP.

(IX) Find RNAP holoenzyme dynamics common to *spo0A* and non-*spo0A* promoters

In Result Section 2.5, to verify whether σ^A- and σ^H-RNAP holoenzyme levels are time-dependent in general, we measured the activity of two non-*spo0A* promoters: P*hyper-spac* (σ^A specific) and *citG* promoter P*citG* (σ^H specific) over 7 hours. Below we show the method adopted to find a

common time-dependent σ^{A}-RNAP increase that explains both P*hyper-spac* and P*v* gene expression. The same method can be applied to find the σ^{H}-RNAP increase for P*citG* and P*s*.

To start with, we assume the increase in σ^{A}-RNAP is identical between the 'P*v* strain, none' expression and the constitutive expression of P*hyper-spac*. Since both strains do not include 0A boxes, these strains allow us to focus on the effect of change in σ^{A}-RNAP level solely. Based on the same modeling framework for a single promoter (Supplementary Section II), there are only two binding configurations possible for such DNA fragment with one promoter (i.e., $[\sigma_1 = 0, \sigma_2 = 0, \sigma_3 = 0, \sigma_{pro} = 0]$ and $[\sigma_1 = 0, \sigma_2 = 0, \sigma_3 = 0, \sigma_{pro} = 1]$). Thus, the effective transcription rate of each promoter, contributed by the promoter-bound configuration, can be written using *Eqn. II. 2*:

$$v_{none_{one\,promoter}}^{effective^h} = v_{max}^{h} P^{h} \qquad \textit{Eqn. IX. 1}$$

$$v_{none_{one\,promoter}}^{effective^v} = v_{max}^{v} P^{v} \qquad \textit{Eqn. IX. 2}$$

Here, the superscript 'h' or 'v' denotes promoter-specific maximum transcription initiation rate and promoter-specific RNAP binding probability, for the configuration $[\sigma_1 = 0, \sigma_2 = 0, \sigma_3 = 0, \sigma_{pro} = 1]$ in each promoter.

The RNAP binding probability of each promoter can be written using *Eqn. II. 5* and *Eqn. II. 6* as:

$$P^{h} = \frac{\exp\left(-G^{\circ h}_{pro}\right) \times [R^{h}]}{\exp\left(-G^{\circ h}_{pro}\right) \times [R^{h}] + 1} \qquad \textit{Eqn. IX. 3}$$

$$P^{v} = \frac{\exp\left(-G^{\circ v}_{pro}\right) \times [R^{v}]}{\exp\left(-G^{\circ v}_{pro}\right) \times [R^{v}] + 1} \qquad \textit{Eqn. IX. 4}$$

where, $G^{\circ h}_{pro}$ and $G^{\circ v}_{pro}$ are RNAP binding affinity to the *hyspac and spo0A* vegetative promoter, respectively. Here, there is no binding of transcription factor such as 0A~P (i.e., $[0A{\sim}P] = 0$).

In accordance with the assumption in the RNAP holoenzyme, we rename the RNAP concentration variable as $[R^{common}]$:

$$[R^{h}] = [R^{v}] = [R^{common}]$$

By examining *Eqn. IX. 1-4,* one can see that there are two parameters (i.e., v_{max} and G_{pro}) that can be potentially responsible for the difference in effective transcription rate between P*hyspac*

and P*v* (i.e., between $v_{none_{one\,promoter}}^{effective^h}$ and $v_{none_{one\,promoter}}^{effective^v}$). Here, we assume that the maximum transcription initiation rate is the same between the promoters (renamed as $v_{max}^h = v_{max}^v = v_{max}^{common}$), but the binding affinity of each promoter is unique ($G_{pro}^{\circ h} \neq G_{pro}^{\circ v}$).

To this end, let the gene expression of P*hyspac* and P*v* at a particular time point $t = w$ hour be $v_{none_{one\,promoter}}^{effective^h}|_{t=w}$ and $v_{none_{one\,promoter}}^{effective^v}|_{t=w}$, and let the RNAP holoenzyme concentration at the same time point be $[R]_{t=w}$. As such, the ratio of model-predicted effective transcription rate between two promoters at $t = w$ hour can be expressed as:

$$\frac{v_{none_{one\,promoter}}^{effective^h}|_{t=w}}{v_{none_{one\,promoter}}^{effective^v}|_{t=w}} = \frac{v_{max}^{common}\dfrac{\exp(-G_{pro}^{\circ h}) \times [R^{common}]_{t=w}}{\exp(-G_{pro}^{\circ h}) \times [R^{common}]_{t=w} + 1}}{v_{max}^{common}\dfrac{\exp(-G_{pro}^{\circ v}) \times [R^{common}]_{t=w}}{\exp(-G_{pro}^{\circ v}) \times [R^{common}]_{t=w} + 1}} = \frac{\dfrac{[R^{common}]_{t=w}}{[R^{common}]_{t=w} + \dfrac{1}{\exp(-G_{pro}^{\circ h})}}}{\dfrac{[R^{common}]_{t=w}}{[R^{common}]_{t=w} + \dfrac{1}{\exp(-G_{pro}^{\circ v})}}} \qquad \text{Eqn. IX. 5}$$

Next, we define the ratio in experimentally measured promoter activity between two promoters at $t = w$ hour as $\frac{X^{real^h}|_{t=w}}{X^{real^v}|_{t=w}}$

To find the σ^A-RNAP increase that explains both P*hyper-spac* and P*v* gene expression, we minimize the difference (E_{sigma}) between model-predicted and measured ratio of gene expression across all time points:

$$E_{sigma} = \frac{1}{7}\sum_{t\in[2,8]} \frac{\dfrac{v_{none_{one\,promoter}}^{effective^h}|_{t=w}}{v_{none_{one\,promoter}}^{effective^v}|_{t=w}} - \dfrac{X^{real^h}|_{t=w}}{X^{real^v}|_{t=w}}}{\dfrac{X^{real^h}|_{t=w}}{X^{real^v}|_{t=w}}} \qquad \text{Eqn. IX. 6}$$

We found reasonable $G_{pro}^{\circ h}$, $G_{pro}^{\circ w}$, and $[R^{common}]_{t=w}$ ($i \in [2,8]$) parameters using MATLAB (2021a) Particle Swarm Optimization (2).

Supplementary Tables

Table S1. List of *lacZ* reporter fusion strains used.. S32

Table S2. Optimization error of the thermodynamic model fit to EMSA data under different assumptions of cooperativity S34

Table S3. List of the parameters used in the biophysical models.................................... S34

Table S4. Model-predicted parameter values from the best fit purely kinetic model with the existence of all higher-order interaction energies.. S37

Table S5. The optimization error of the purely kinetic biophysical model under different assumptions on the existence of high-order interaction energy parameters.................. S39

Table S6. Dissection of the effect of 0A~P binding and RNAP binding on *spo0A* expression for each mutant strain S41

Table S1. List of *lacZ* reporter fusion strains used

Also shown is the 'Alias' name used to refer to each strain in the main manuscript and supplementary files.

B. subtilis **strains identifier**	**Reference**	**Genotype**	**Alias**
amyE::Pspo0A^{wt}-lacZ spc comI^{Q12L} (9766 → 5609) S	(1)	No Pv or Ps removal, no 0A mutation	P*v*P*s* strain, 123
amyE::Pspo0A^{0A1*}-lacZ spc comI^{Q12L} (9673 → 5609) S	(1)	No Pv or Ps removal, 0A1 mutated	P*v*P*s* strain, 23
amyE::Pspo0A^{0A2*}-lacZ spc comI^{Q12L} (9674 → 5609) S	(1)	No Pv or Ps removal, 0A2 mutated	P*v*P*s* strain, 13
amyE::Pspo0A^{0A3*}-lacZ spc comI^{Q12L} (9675 → 5609) S	(1)	No Pv or Ps removal, 0A3 mutated	P*v*P*s* strain, 12
amyE::Pspo0A^{0A1*2*}-lacZ spc comI^{Q12L} (pMF1056 → 5609) S	(1)	No Pv or Ps removal, 0A1 and 0A2 mutated	P*v*P*s* strain, 3
amyE::Pspo0A^{0A1*3*}-lacZ spc comI^{Q12L} (pMF1010 → 5609) S	(1)	No Pv or Ps removal, 0A1 and 0A3 mutated	P*v*P*s* strain, 2
amyE::Pspo0A^{0A2*3*}-lacZ spc comI^{Q12L} (pMF1057 → 5609) S	(1)	No Pv or Ps removal, 0A2 and 0A3 mutated	P*v*P*s* strain, 1
amyE::Pspo0A^{0A1*2*3*}-lacZ spc comI^{Q12L} (pMF1058 → 5609) S	(1)	No Pv or Ps removal, 0A1, 0A2, 0A3 mutated	P*v*P*s* strain, none
amyE::Pspo0A^{0A4*}-lacZ spc comI^{Q12L} (10128 → 5609) S	(1)	Ps removed, no 0A mutation	P*v* strain, 123
amyE::Pspo0A^{0A1*4*}-lacZ spc comI^{Q12L} (10129 → 5609) S	(1)	Ps removed, 0A1 mutated	P*v* strain, 23
amyE::Pspo0A^{0A2*4*}-lacZ spc comI^{Q12L} (10130 → 5609) S	(1)	Ps removed, 0A2 mutated	P*v* strain, 13
amyE::Pspo0A^{0A3*4*}-lacZ spc comI^{Q12L} (10131 → 5609) S	(1)	Ps removed, 0A3 mutated	P*v* strain, 12
amyE::Pspo0A^{0A1*2*4*}-lacZ spc comI^{Q12L} (pMF1059 →5609) S	(1)	Ps removed, 0A1 and 0A2 mutated	P*v* strain, 3
amyE::Pspo0A^{0A1*3*4*}-lacZ spc comI^{Q12L} (pMF1043 → 5609) S	(1)	Ps removed, 0A1 and 0A3 mutated	P*v* strain, 2
amyE::Pspo0A^{0A2*3*4*}-lacZ spc comI^{Q12L} (pMF1060 →5609) S	(1)	Ps removed, 0A2 and 0A3 mutated	P*v* strain, 1

amyE::Pspo0A^{0A1*2*3*4*}-lacZ spc comI^{Q12L} (pMF1061 →5609) S	(1)	Ps removed, 0A1, 0A2, 0A3 mutated	P*v* strain, none
amyE::$P_{\Delta v}^{wt}$-lacZ spc comI^{Q12L} (9795 → 5609) S	(1)	Pv removed, no 0A mutation	P*s* strain, 123
amyE::$P_{\Delta v}^{0A1*}$-lacZ spc comI^{Q12L} (pMF1068 → 5609) S	(1)	Pv removed, 0A1 mutated	P*s* strain, 23
amyE::$P_{\Delta v}^{0A2*}$-lacZ spc comI^{Q12L} (9796 → 5609) S	(1)	Pv removed, 0A2 mutated	P*s* strain, 13
amyE::$P_{\Delta v}^{0A3*}$-lacZ spc comI^{Q12L} (9797 → 5609) S	(1)	Pv removed, 0A3 mutated	P*s* strain, 12
amyE::$P_{\Delta v}^{0A1*2*}$-lacZ spc comI^{Q12L} (pMF1070 → 5609) S	(1)	Pv removed, 0A1 and 0A2 mutated	P*s* strain, 3
amyE::$P_{\Delta v}^{0A1*3*}$-lacZ spc comI^{Q12L} (pMF1071 → 5609) S	(1)	Pv removed, 0A1 and 0A3 mutated	P*s* strain, 2
amyE::$P_{\Delta v}^{0A2*3*}$-lacZ spc comI^{Q12L} (pMF1072 → 5609) S	(1)	Pv removed, 0A2 and 0A3 mutated	P*s* strain, 1
amyE::$P_{\Delta v}^{0A1*2*3*}$-lacZ spc comI^{Q12L} (pMF1073 →5609) S	(1)	Pv removed, 0A1, 0A2, 0A3 mutated	P*s* strain, none
amyE::Phy-spac-lacZ cm comI ^{Q12L} (342 → 5609) S	Original to this study	Constitutive σ^A specific *hyper-spac* promoter	P*hyper-spac*
amyE::PcitG-lacZ spc comI ^{Q12L} (877 → 5609) C	Original to this study	Constitutive σ^H specific *citG* promoter	P*citG*

Table S2. Optimization error of the thermodynamic model fit to EMSA data under different assumptions of cooperativity

The fold change in optimization error (Supplementary Section I, subsection i.3) between the two optimizations with different N is ~3.7 fold.

Cooperativity	Optimization error
2	0.1410
4	0.0382

Table S3. List of the parameters used in the biophysical models

Parameters	Description
$G^{\circ}{}_{1}$	Binding energy of 0A~P to 0A1
$G^{\circ}{}_{2}$	Binding energy of 0A~P to 0A2
$G^{\circ}{}_{3}$	Binding energy of 0A~P to 0A3
$G^{\circ}{}_{v}$	Binding energy of RNAP to *Pv* promoter
$G^{\circ}{}_{s}$	Binding energy of RNAP to *Ps* promoter
$G^{\circ}{}_{12}$	Interaction energy between 0A~P bound at 0A1 and 0A2
$G^{\circ}{}_{13}$	Interaction energy between 0A~P bound at 0A1 and 0A3
$G^{\circ}{}_{23}$	Interaction energy between 0A~P bound at 0A2 and 0A3
$G^{\circ}{}_{123}$	Interaction energy between 0A~P bound at 0A1, 0A2, and 0A3
$[0A{\sim}P]_{t=w}$	Concentration of 0A~P at the wth hour. $w \in [2,9]$
$[R^{s}]_{t=w}$	Concentration of sigma H-associated RNAP at the wth hour. $w \in [2,9]$
$[R^{v}]_{t=w}$	Concentration of sigma A-associated RNAP at the wth hour. $w \in [2,9]$

$G^{\circ v}_{1pro}$	Interaction energy between 0A~P bound at 0A1 and RNAP bound at *Pv*
$G^{\circ v}_{2pro}$	Interaction energy between 0A~P bound at 0A2 and RNAP bound at *Pv*
$G^{\circ v}_{3pro}$	Interaction energy between 0A~P bound at 0A3 and RNAP bound at *Pv*
$G^{\circ v}_{12pro}$	Interaction energy between 0A~P bound at 0A1, 0A~P bound at 0A2, and RNAP bound at *Pv*
$G^{\circ v}_{13pro}$	Interaction energy between 0A~P bound at 0A1, 0A~P bound at 0A3, and RNAP bound at *Pv*
$G^{\circ v}_{23pro}$	Interaction energy between 0A~P bound at 0A2, 0A~P bound at 0A3, and RNAP bound at *Pv*
$G^{\circ v}_{123pro}$	Interaction energy between 0A~P bound at 0A1, 0A~P bound at 0A2, 0A~P bound at 0A3, and RNAP bound at *Pv*
$v^{v}_{max_{0001}}$	Maximum transcription initiation rate for configurations where *Pv* is bound but no 0A box is bound
$v^{v}_{max_{0011}}$	Maximum transcription initiation rate for configurations where *Pv* is bound and 0A3 is bound
$v^{v}_{max_{0101}}$	Maximum transcription initiation rate for configurations where *Pv* is bound and 0A2 is bound
$v^{v}_{max_{0111}}$	Maximum transcription initiation rate for configurations where *Pv* is bound, 0A2 and 0A3 are bound
$v^{v}_{max_{1001}}$	Maximum transcription initiation rate for configurations where *Pv* is bound and 0A1 is bound
$v^{v}_{max_{1011}}$	Maximum transcription initiation rate for configurations where *Pv* is bound, and 0A1 and 0A3 are bound
$v^{v}_{max_{1101}}$	Maximum transcription initiation rate for configurations where Pv is bound, and 0A1 and 0A2 are bound
$v^{v}_{max_{1111}}$	Maximum transcription initiation rate for configurations where *Pv* is bound, and 0A1, 0A2, and 0A3 are bound

$G^{\circ s}_{1pro}$	Interaction energy between 0A~P bound at 0A1 and RNAP bound at *Ps*
$G^{\circ s}_{2pro}$	Interaction energy between 0A~P bound at 0A2 and RNAP bound at *Ps*
$G^{\circ s}_{3pro}$	Interaction energy between 0A~P bound at 0A3 and RNAP bound at *Ps*
$G^{\circ s}_{12pro}$	Interaction energy between 0A~P bound at 0A1, 0A~P bound at 0A2, and RNAP bound at *Ps*
$G^{\circ s}_{13pro}$	Interaction energy between 0A~P bound at 0A1, 0A~P bound at 0A3, and RNAP bound at *Ps*
$G^{\circ s}_{23pro}$	Interaction energy between 0A~P bound at 0A2, 0A~P bound at 0A3, and RNAP bound at *Ps*
$G^{\circ s}_{123pro}$	Interaction energy between 0A~P bound at 0A1, 0A~P bound at 0A2, 0A~P bound at 0A3, and RNAP bound at *Ps*
$v^{s}_{max_{0001}}$	Maximum transcription initiation rate for configurations where *Ps* is bound but no 0A box is bound
$v^{s}_{max_{0011}}$	Maximum transcription initiation rate for configurations where *Ps* is bound and 0A3 is bound
$v^{s}_{max_{0101}}$	Maximum transcription initiation rate for configurations where *Ps* is bound and 0A2 is bound
$v^{s}_{max_{0111}}$	Maximum transcription initiation rate for configurations where *Ps* is bound and 0A2 and 0A3 are bound
$v^{s}_{max_{1001}}$	Maximum transcription initiation rate for configurations where *Ps* is bound and 0A1 are bound
$v^{s}_{max_{1011}}$	Maximum transcription initiation rate for configurations where *Ps* is bound, and 0A1 and 0A3 are bound
$v^{s}_{max_{1101}}$	Maximum transcription initiation rate for configurations where *Ps* is bound, and 0A1 and 0A2 are bound
$v^{s}_{max_{1111}}$	Maximum transcription initiation rate for configurations where Ps is bound, and 0A1, 0A2, and 0A3 are bound

Table S4. Model-predicted parameter values from the best fit purely kinetic model with the existence of all higher-order interaction energies

Higher-order interaction energies refer to G°_{12}, G°_{13}, G°_{23}, and G°_{123} in the biophysical model. The mean of predicted parameters across 20 independent optimization runs is reported with standard deviation. Full parameter list and corresponding descriptions are shown in Table S3. Note that the energies between 0A boxes and promoters do not exist (values are zero, thus not shown) in the purely kinetic biophysical model. Values of G°_{1}, G°_{2}, G°_{3} are not directly optimized, but obtained by offsetting $G_{EMSA_1}, G_{EMSA_2}, G_{EMSA_3}$ with a single, fitted parameter (Supplementary Section V).

Parameter	Mean	Std	Unit
G°_{1}	-5.42	<10e-3	kT
G°_{2}	-2.35	<10e-3	kT
G°_{3}	-7.40	<10e-3	kT
G°_{12}	-8.54	1.22	kT
G°_{13}	-8.82	5.04	kT
G°_{23}	-13.96	0.087	kT
G°_{123}	-9.58	7.0283	kT
$v^{v}_{max_{0001}}$	685.03	22.17	a.u.
$v^{v}_{max_{0011}}$	617.18	13.80	a.u.
$v^{v}_{max_{0101}}$	628.38	11.17	a.u.
$v^{v}_{max_{0111}}$	693.85	17.22	a.u.
$v^{v}_{max_{1001}}$	410.64	7.77	a.u.
$v^{v}_{max_{1011}}$	412.87	11.94	a.u.
$v^{v}_{max_{1101}}$	350.20	5.93	a.u.

$v^{v}_{max_{1111}}$	466.28	14.93	a.u.
$v^{s}_{max_{0001}}$	977.09	34.19	a.u.
$v^{s}_{max_{0011}}$	1254.91	41.21	a.u.
$v^{s}_{max_{0101}}$	736.31	23.62	a.u.
$v^{s}_{max_{0111}}$	149.61	5.15	a.u.
$v^{s}_{max_{1001}}$	1077.36	34.68	a.u.
$v^{s}_{max_{1011}}$	1233.93	40.93	a.u.
$v^{s}_{max_{1101}}$	680.73	23.31	a.u.
$v^{s}_{max_{1111}}$	280.15	10.18	a.u.
$[R^{v}]_{t=2}$	0.25	0.01	µM
$[R^{v}]_{t=3}$	0.35	0.02	µM
$[R^{v}]_{t=4}$	0.35	0.01	µM
$[R^{v}]_{t=5}$	0.50	0.02	µM
$[R^{v}]_{t=6}$	0.73	0.03	µM
$[R^{v}]_{t=7}$	1.11	0.06	µM
$[R^{v}]_{t=8}$	1.37	0.08	µM
$[R^{v}]_{t=9}$	2.00	0.02	µM
$[R^{s}]_{t=2}$	0.06	0.002	µM
$[R^{s}]_{t=3}$	0.13	0.005	µM
$[R^{s}]_{t=4}$	0.44	0.02	µM
$[R^{s}]_{t=5}$	0.73	0.05	µM

$[R^s]_{t=6}$	1.08	0.07	µM
$[R^s]_{t=7}$	1.33	0.09	µM
$[R^s]_{t=8}$	1.73	0.14	µM
$[R^s]_{t=9}$	1.90	0.15	µM

Table S5. The optimization error of the purely kinetic biophysical model under different assumptions on the existence of high-order interaction energy parameters

The optimization error is computed based on Material and Methods Section 4.5, *Eqn. 5*. Higher-order interaction energies refer to $G^{\circ}{}_{12}$, $G^{\circ}{}_{13}$, $G^{\circ}{}_{23}$, and $G^{\circ}{}_{123}$ in the biophysical model. Shown in the second column is, under the current assumption, how many higher-order interaction energies are nonzero (i.e., the energy exists). The Bayesian information criterion (BIC) was computed for each assumption. In grey is the best fitting model (i.e. lowest optimization error), where all secondary energy parameters are nonzero and tertiary energy parameter is zero. Full parameter list and descriptions are shown in Table S3.

**Assumption for energy parameters**	**Number of higher-order energies being nonzero**	**Optimization error**	**BIC**
$G^{\circ}{}_{12} = 0$, $G^{\circ}{}_{13} = 0$, $G^{\circ}{}_{23} = 0$, $G^{\circ}{}_{123} = 0$	0	0.1886	-0.7646
$G^{\circ}{}_{12} \neq 0$, $G^{\circ}{}_{13} = 0$, $G^{\circ}{}_{23} = 0$, $G^{\circ}{}_{123} = 0$	1	0.1665	-0.8619
$G^{\circ}{}_{12} = 0$, $G^{\circ}{}_{13} \neq 0$, $G^{\circ}{}_{23} = 0$, $G^{\circ}{}_{123} = 0$	1	0.1807	-0.7799
$G^{\circ}{}_{12} = 0$, $G^{\circ}{}_{13} = 0$, $G^{\circ}{}_{23} \neq 0$, $G^{\circ}{}_{123} = 0$	1	0.1375	-1.0533
$G^{\circ}{}_{12} = 0$, $G^{\circ}{}_{13} = 0$, $G^{\circ}{}_{23} = 0$, $G^{\circ}{}_{123} \neq 0$	1	0.1740	-0.8176

$G°_{12} \neq 0$, $G°_{13} \neq 0$, $G°_{23} = 0$, $G°_{123} = 0$	2	0.1538	-0.9140
$G°_{12} \neq 0$, $G°_{13} = 0$, $G°_{23} \neq 0$, $G°_{123} = 0$	2	0.1118	-1.1720
$G°_{12} \neq 0$, $G°_{13} = 0$, $G°_{23} = 0$, $G°_{123} \neq 0$	2	0.1508	-0.9333
$G°_{12} = 0$, $G°_{13} \neq 0$, $G°_{23} \neq 0$, $G°_{123} = 0$	2	0.1286	-1.0929
$G°_{12} = 0$, $G°_{13} \neq 0$, $G°_{23} = 0$, $G°_{123} \neq 0$	2	0.1605	-0.8708
$G°_{12} = 0$, $G°_{13} = 0$, $G°_{23} \neq 0$, $G°_{123} \neq 0$	2	0.1259	-1.1140
$G°_{12} \neq 0$, $G°_{13} \neq 0$, $G°_{23} \neq 0$, $G°_{123} = 0$	3	0.0731	-1.6301
$G°_{12} \neq 0$, $G°_{13} \neq 0$, $G°_{23} = 0$, $G°_{123} \neq 0$	3	0.1369	-1.0026
$G°_{12} \neq 0$, $G°_{13} = 0$, $G°_{23} \neq 0$, $G°_{123} \neq 0$	3	0.0774	-1.5725
$G°_{12} = 0$, $G°_{13} \neq 0$, $G°_{23} \neq 0$, $G°_{123} \neq 0$	3	0.0858	-1.4697
$G°_{12} \neq 0$, $G°_{13} \neq 0$, $G°_{23} \neq 0$, $G°_{123} \neq 0$	4	0.0731	-1.6033

Table S6. Dissection of the effect of 0A~P binding and RNAP binding on *spo0A* expression for each mutant strain

Using best-fit model parameters (Table S4), the biophysical model was simulated with either constant 0A~P concentration (second column) or constant RNAP holoenzyme concentration (third column) over time. For each simulation, the fold change of effective transcription rate in the last time point (t = 9h) in comparison to the first time point (t = 2h) was computed for each entry.

**Mutant Strain**	**Fold increase in $v^{effective}$ due to RNAP holoenzyme only**	**Fold increase in $v^{effective}$ due to 0A~P only**
P*v*, 1	3.4624	0.6102
P*v*, 2	3.4624	0.9050
P*v*, 3	3.4624	0.9293
P*v*, 12	3.4624	0.6831
P*v*, 13	3.4624	0.7881
P*v*, 23	3.4624	1.0000
P*v*, 123	3.4624	0.9993
P*v*, none	3.4624	1.0000
P*s*, 1	11.5727	1.0923
P*s*, 2	11.5727	0.7564
P*s*, 3	11.5727	1.1597
P*s*, 12	11.5727	0.8166
P*s*, 13	11.5727	1.0550
P*s*, 23	11.5727	0.9964
P*s*, 123	11.5727	1.0007
P*s*, none	11.5727	1.0000
P*v*P*s*, 1	5.8806	0.7540
P*v*P*s*, 2	5.8141	0.8619
P*v*P*s*, 3	6.0573	1.0030
P*v*P*s*, 12	6.0642	0.7259
P*v*P*s*, 13	6.6931	0.8944
P*v*P*s*, 23	3.9389	0.9998
P*v*P*s*, 123	4.6452	0.9995
P*v*P*s*, none	5.8151	1.0000

Supplementary Figures

Figure S1. Fitting the experimentally determined fraction of bound DNA to a statistical thermodynamic model with a Hill coefficient (*N*) of 2 .... S43

Figure S2. Predicting the fraction of bound DNA for constructs with multiple boxes present using single binding energies optimized from the statistical thermodynamic model S44

Figure S3. Model input dynamics of Spo0A~P concentration for the wildtype (WT) strain S44

Figure S4. Optimization error for all biophysical models examined S45

Figure S5. Additivity of thermodynamic and kinetic control models S45

Figure S6. Probability that all 0A boxes are bound for strains with one, two, or three boxes on the DNA fragment S46

Figure S7. Model-predicted gene expression for all mutant strains computed with constant sigma factor levels S46

Figure S8. Model-predicted RNAP holoenzyme dynamics S47

Figure S9. Normalized model-predicted effective transcription rate S48

Figure S10. Sigma factor activities in *spo0A* promoter and other promoters S49

Figure S11. Energy diagram of transcription modeled as a two-step process S50

Figure S12. Energy diagram of transcription with purely thermodynamic control S51

Figure S13. Energy diagram of transcription with purely kinetic control S52

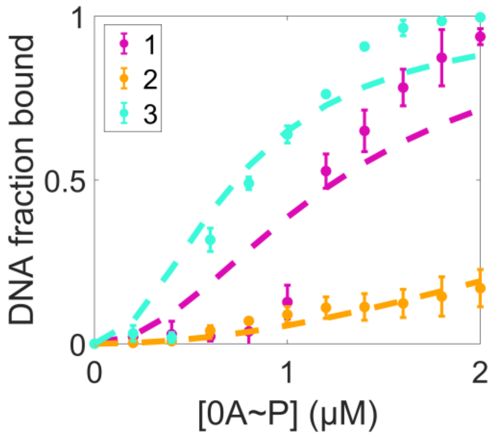

Figure S1. Fitting the experimentally determined fraction of bound DNA to a statistical thermodynamic model with a Hill coefficient (*N*) of 2

Model prediction (dashed lines) and empirical measurement (solid lines with standard deviation error bars) are shown for DNA fragments with one 0A box mutation. The fitting is worse than when considering cooperativity (*N*) of 4.

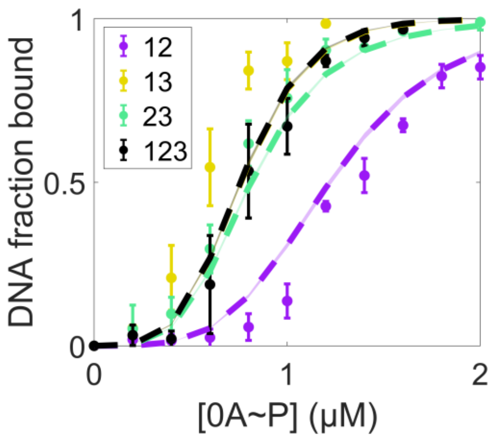

Figure S2. Predicting the fraction of bound DNA for constructs with multiple boxes present using single binding energies optimized from the statistical thermodynamic model

Solid data points with error bars are experimental measurements with standard deviation. Dashed lines with colored patches are mean predictions with standard deviations, using different parameter sets derived from 20 augmented measurements. Dashed yellow and dashed black lines overlap.

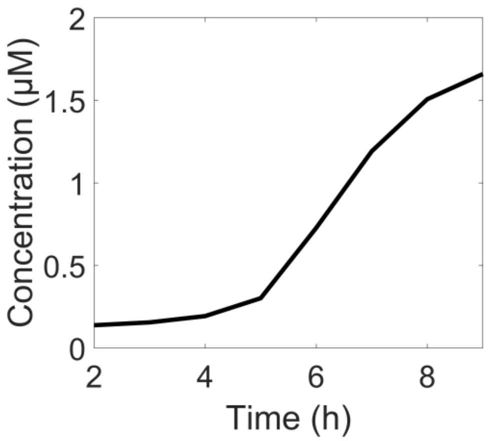

Figure S3. Model input dynamics of Spo0A~P concentration for the wildtype (WT) strain

Predicted dynamics are based on (12).

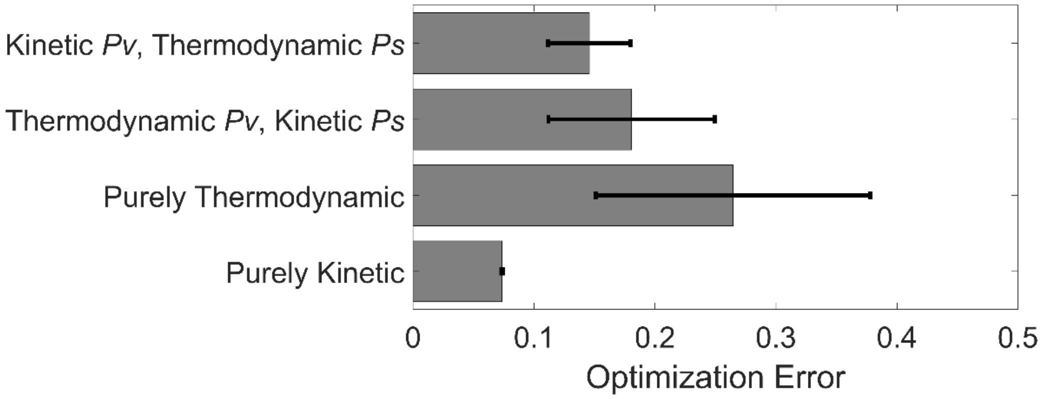

Figure S4. Optimization error for all biophysical models examined

Each model was optimized at least 20 times to examine if it best fits the experimental *spo0A* promoter activity using the MATLAB Particle Swarm Optimization algorithm (2). The number of fitting parameters was kept the same across all models. Shown is the mean and standard deviation of the output of the objective function (Material and Methods Section 4.5, *Eqn. 5*) for the four transcriptional control models tested.

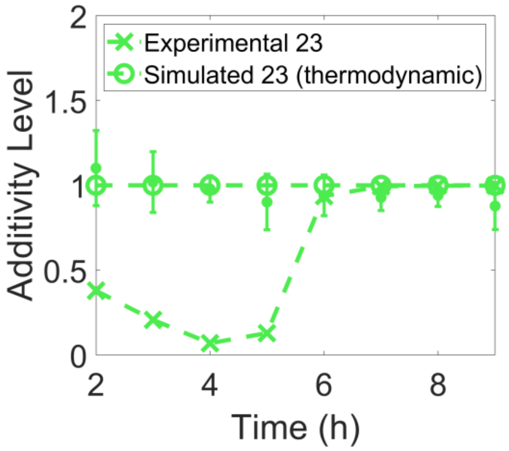

Figure S5. Additivity of thermodynamic and kinetic control models

Additivity level for the purely kinetic model (marker “O”) and for the purely thermodynamic model (marker “X”) with two 0A boxes on the DNA fragment, under randomly sampled energy parameters. The experimental data (solid line with colored regions and error bar) with two 0A boxes (0A2 and 0A3) are shown for reference.

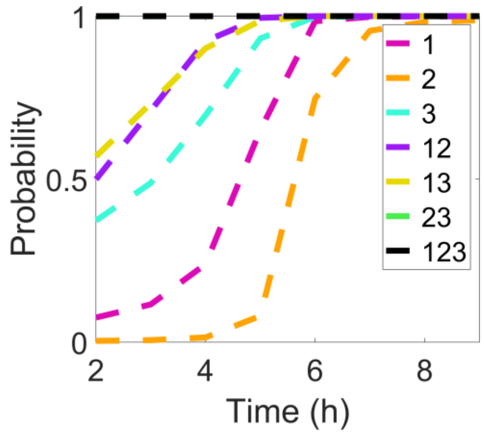

Figure S6. Probability that all 0A boxes are bound for strains with one, two, or three boxes on the DNA fragment

Occupancy probability is computed with 0A~P dynamics shown in Supplementary Figure S3 and best-fit energy parameters in Supplementary Table S4.

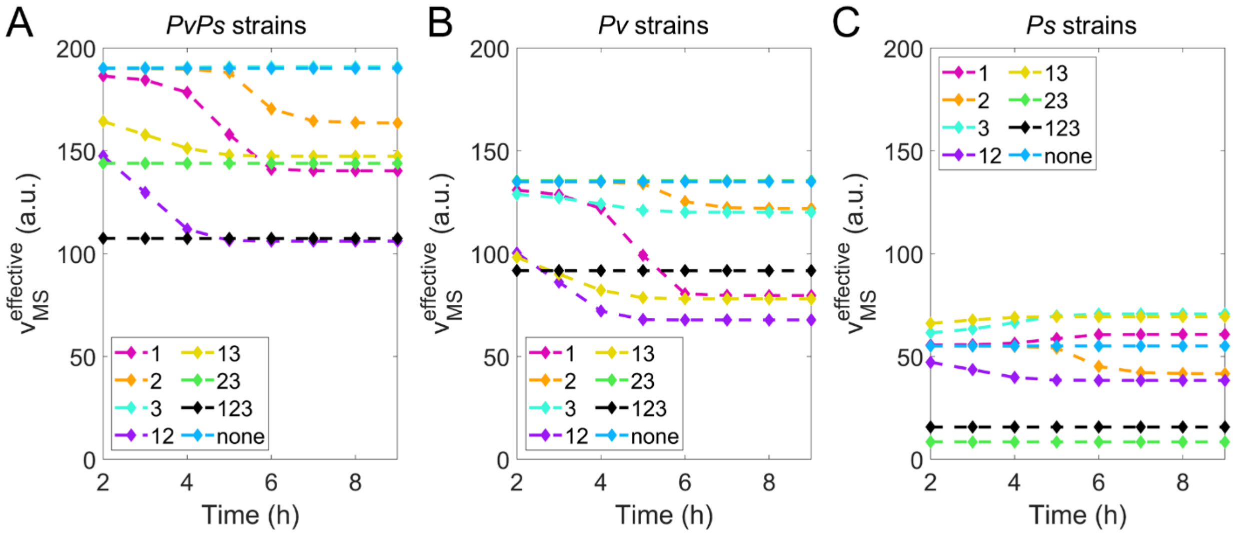

Figure S7. Model-predicted gene expression for all mutant strains computed with constant sigma factor levels

Each plot corresponds to a promoter-specific strain **A)** ‘P*v*P*s* strains’ with constant σ^A-RNAP and constant σ^H-RNAP, **B)** ‘P*v* strains’ with constant σ^A-RNAP and **C)** ‘P*s* strains’ with constant σ^H-RNAP.

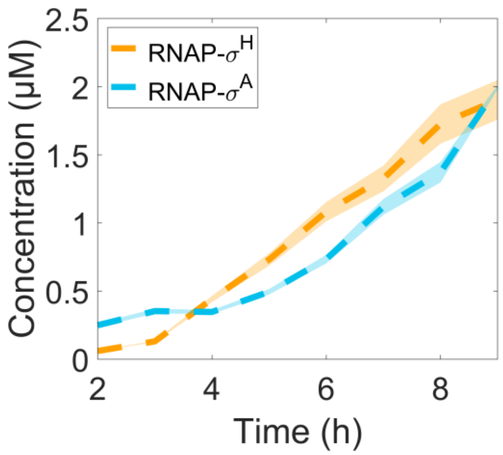

Figure S8. Model-predicted RNAP holoenzyme dynamics

Dashed lines are the mean concentration of RNAP holoenzyme bearing σ^A (blue) and σ^H (yellow). Colored patches are the standard deviations in predicted dynamics across 20 independent optimizations based on Table S4.

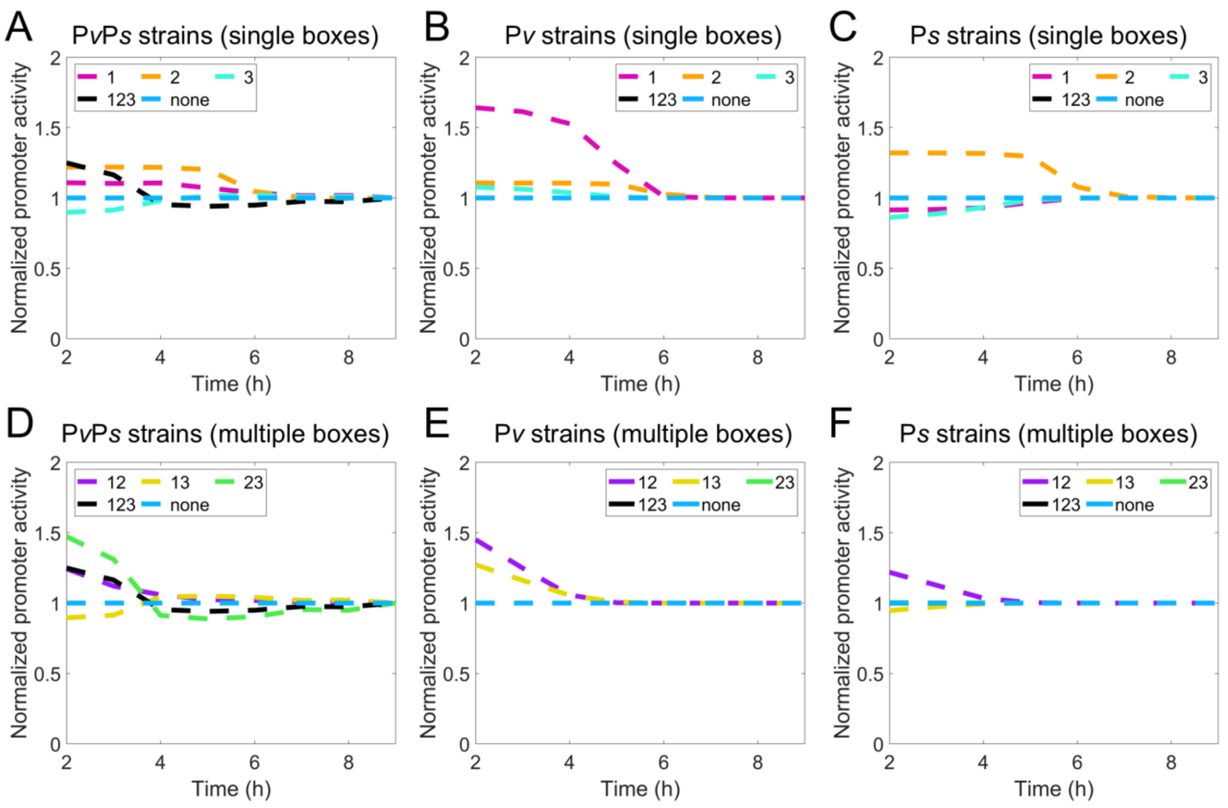

Figure S9. Normalized model-predicted effective transcription rate

Shown are normalized v^{eff} for strains with **(A-C)** single boxes and with **(D-F)** multiple boxes. Note the normalization is done by dividing the time-dependent prediction by its own final-time prediction, then by the prediction of 'none' for each promoter-specific construct. Normalization is performed for all dashed lines in Figure 5B-G.

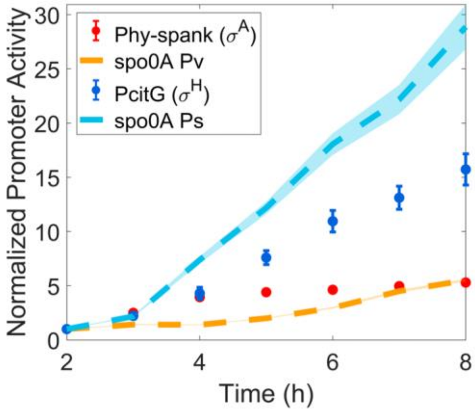

Figure S10. Sigma factor activities in *spo0A* promoter and other promoters

Normalized expression of two σ^{A}-dependent promoter over time, *hyper-spac* and P*v* of *spo0A*; also shown is the normalized expression of two σ^{H}-dependent promoters over time, *citG* and P*s* of *spo0A*. Note that data normalization is done by dividing each measurement by its corresponding measurement at t = 2 hours. Expression of *spo0A* P*v* and *spo0A* P*s* is the same as 'P*v* strain, none' and 'P*s* strain, none', respectively. The non-overlapping between blue dashed line and blue filled circles indicates *hyper-spac* and P*v* have different promoter binding affinity. The same is true for *citG* and P*s.*

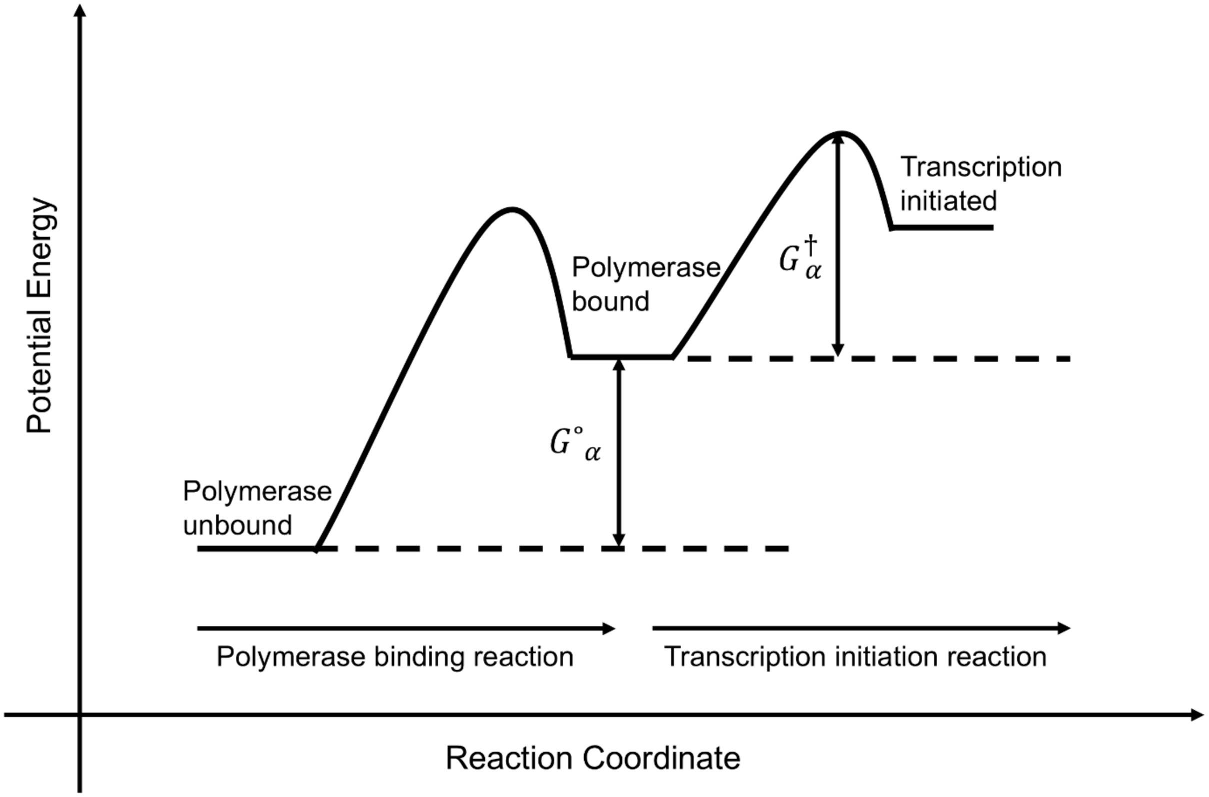

Figure S11. Energy diagram of transcription modeled as a two-step process

Reaction 1 corresponds to RNAP binding to the promoter (closed-complex formation). Reaction 2 corresponds to the post-closed-complex step. Namely, isomerization into an open complex and RNA synthesis initiation (8). Activation energy, $G°_{\alpha}$, between product and reactant state of reaction 1 determines the probability of RNAP binding (*Eqn. II. 4*). Activation energy of reaction 2, $G_{\alpha}^{\dagger}$, determines the maximum rate of transcription $v_{max_{\alpha}}$ (*Eqn. II. 10*).

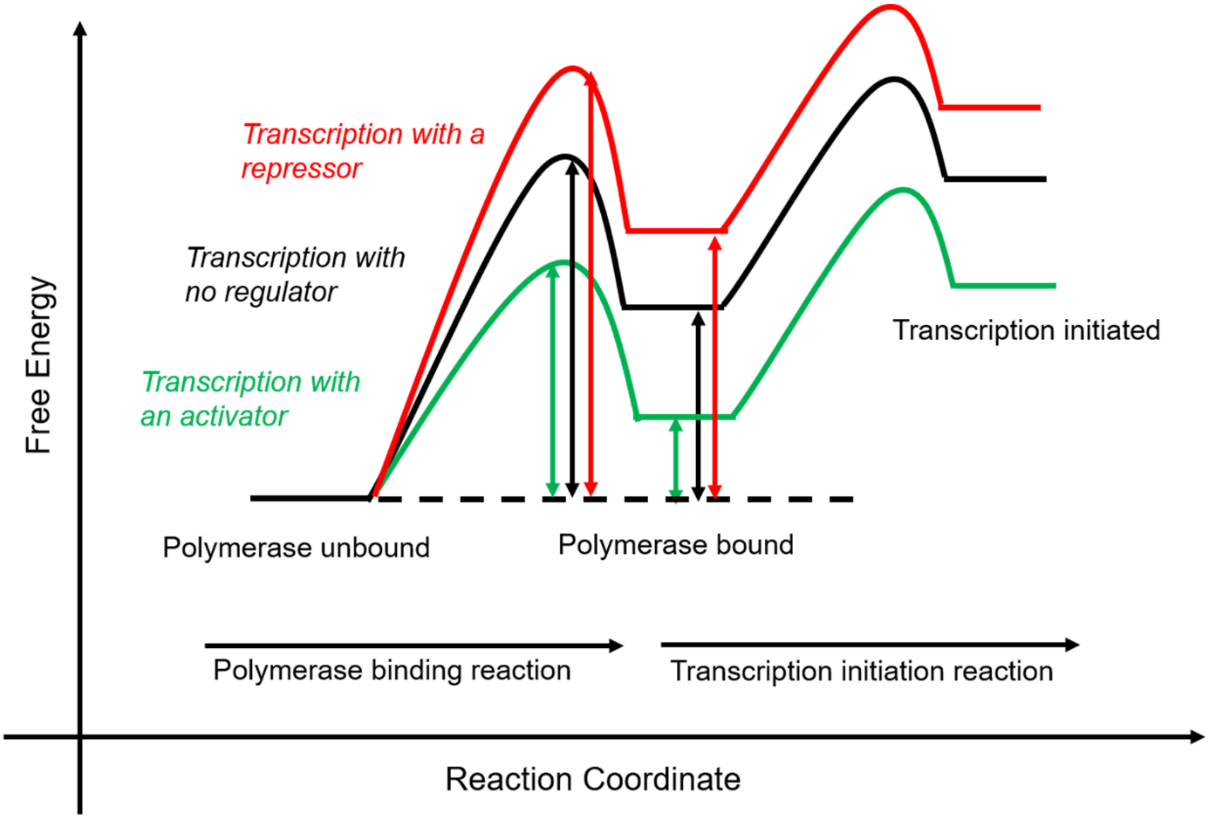

Figure S12. Energy diagram of transcription with purely thermodynamic control

Upon binding of the transcriptional factor, an activator (repressor) lowers (increases) the potential energy of the polymerase binding reaction, indirectly lowering (increasing) the activation barrier in the same reaction, but there is no change in the activation energy of the transcription initiation reaction.

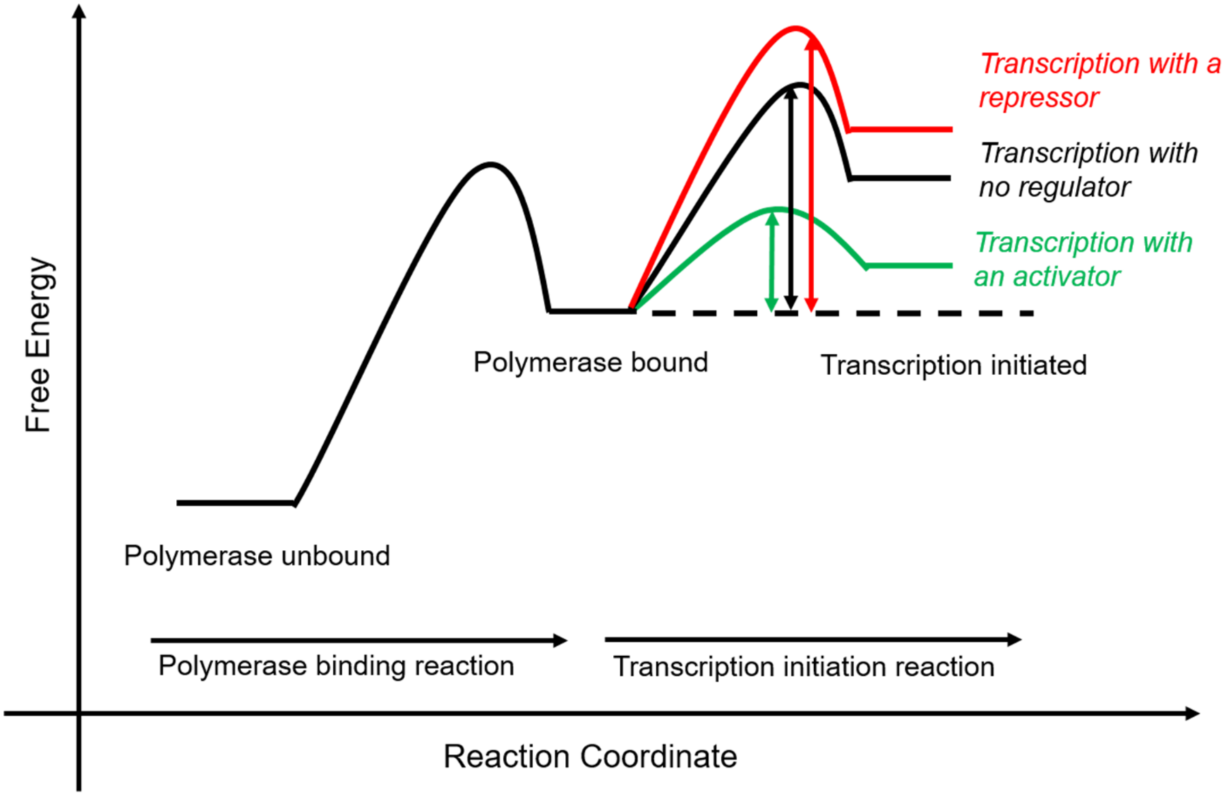

Figure S13. Energy diagram of transcription with purely kinetic control

Upon binding of the transcriptional factor, an activator (repressor) lowers (increases) the activation energy of the transcription initiation reaction, but there is no change in the free energy of the polymerase binding reaction.

SI References

1. Zarazúa-Osorio B, Srivastava P, Marathe A, Zahid SH, Fujita M. 2025. Autoregulation of the Master Regulator Spo0A Controls Cell-Fate Decisions in *Bacillus subtilis*. Mol Microbiol https://doi.org/10.1111/mmi.15341.

2. Yarpiz / Mostapha Heris. Particle swarm optimization (PSO) (2021a). MATLAB. MATLAB Central File Exchange.

3. Ackers GK, Johnson AD, Shea MA. 1982. Quantitative model for gene regulation by lambda phage repressor. Proc Natl Acad Sci 79:1129–1133.

4. Morrison M, Razo-Mejia M, Phillips R. 2021. Reconciling kinetic and thermodynamic models of bacterial transcription. PLOS Comput Biol 17:e1008572.

5. Shea MA, Ackers GK. 1985. The OR control system of bacteriophage lambda. A physical-chemical model for gene regulation. J Mol Biol 181:211–230.

6. Igoshin OA, Narula J. 2010. Thermodynamic models of combinatorial gene regulation by distant enhancers. IET Syst Biol 4:393–408.

7. Narula J, Smith AM, Gottgens B, Igoshin OA. 2010. Modeling reveals bistability and low-pass filtering in the network module determining blood stem cell fate. PLoS Comput Biol 6:e1000771.

8. McClure WR. 1985. Mechanism and control of transcription initiation in prokaryotes. Annu Rev Biochem 54:171–204.

9. Saecker RM, Record MT, deHaseth PL. 2011. Mechanism of bacterial transcription initiation: RNA polymerase - promoter binding, isomerization to initiation-competent open complexes, and initiation of RNA synthesis. J Mol Biol 412:754–771.

10. Bintu L, Buchler NE, Garcia HG, Gerland U, Hwa T, Kondev J, Phillips R. 2005. Transcriptional regulation by the numbers: models. Curr Opin Genet Dev 15:116–124.

11. Schwarz G. 1978. Estimating the dimension of a model. Ann Stat 6:461–64.

12. Chen Z, Srivastava P, Zarazúa-Osorio B, Marathe A, Fujita M, Igoshin OA. 2022. *Bacillus subtilis* histidine kinase KinC activates biofilm formation by controlling heterogeneity of single-cell responses. mBio 13:e01694-21.
